# Phenotypic and metabonomics studies of FMOs in *C. elegans* and their roles in lifespan extension

**DOI:** 10.1007/s11306-025-02367-4

**Published:** 2025-11-15

**Authors:** Mohamed Said, Rafael Freire, Filipe Cabreiro, Jose Ivan Serrano-Contreras, Elinor P. Thompson, Jeremy R. Everett

**Affiliations:** 1 Faculty of Engineering and Science, University of Greenwich, Chatham Maritime, Kent, ME4 4TB UK; 2 Institute of Clinical Sciences, Imperial College London, London, W12 0NN UK; 3 Cologne Excellence Cluster for Cellular Stress Responses in Ageing-Associated Diseases (CECAD), University of Cologne, Joseph Stelzmann Strasse 26, 50931 Cologne, Germany; 4 Department of Metabolism, Faculty of Medicine, Digestion and Reproduction, Commonwealth Building, Hammersmith Campus, Imperial College, Du Cane Road, London, W12 0NN UK; 5 Present Address: Faculty of Pharmacy, MSA University, 6th October City, Giza Egypt; 6 Present Address: BASF SE, RGA/AL, Carl-Bosch-Strasse 38, B007-0543, 67056 Ludwigshafen am Rhein, Germany

**Keywords:** *C. elegans*, FMO Genotypes, Metabonomics, Metabolomics, NMR Spectroscopy, Lifespan

## Abstract

**Introduction:**

Flavin-Containing Monooxygenases (FMO) are widely conserved, xenobiotic-detoxifying enzymes whose additional endogenous functions have been revealed in recent studies. Those roles include the regulation of longevity in the model nematode *Caenorhabditis elegans.*

**Objectives:**

The purpose of this study was to compare aspects of the phenotypes of *C. elegans* worms with mutations in all *fmo* genes, particularly focusing on the metabolome and its relationship with lifespan-extension and the worm life cycle. This is the first systematic study of the effect of *fmo* genetic variation on *C. elegans* metabolic profiles that we are aware of.

**Methods:**

NMR Spectroscopic analysis of the extracts of metabolites from *C. elegans* worms of different ages and *fmo* genotypes was used to compare metabolite profiles of *C. elegans* worms and determine how these changed with genotype and ageing.

**Results:**

Loss of both *fmo-4* and *fmo-3* and over-expression of *fmo-*2, resulted in increased levels of tryptophan in the metabolome, which correlated with an extended lifespan in these mutants. Loss of *fmo-4* also led to decreased embryo hatching, along with increased sensitivity to bleach during sterilisation protocols. In contrast, in the extended lifespan *fmo-1* knockout worm, the metabolome did not reveal any significant metabolite changes and therefore lifespan effects may occur through another mechanism, or hidden metabolic changes.

**Conclusion:**

Genetic interventions coupled with metabolome profiling in *C. elegans* can provide insights into biological mechanisms in ageing that might lead to strategies for healthy lifespan extension in human old age.

**Supplementary Information:**

The online version contains supplementary material available at 10.1007/s11306-025-02367-4.

## Introduction

Ageing is a progressive and intrinsic biological degenerative process in the body, with accumulated molecular deterioration in many tissues and cellular pathways. This biochemical decline over time leads to cellular damage and dysfunction and, finally, to death (DiLoreto & Murphy, 2015; Bratic & Larsson, 2013; Leiser et al., 2015; López-Otín et al., 2023). One of the key drivers in 21 st century healthcare research is the extension of healthy lifespan in an ageing population (Mount et al., 2016). As part of these efforts to enhance quality of life and healthy ageing, it is desirable to reduce the risk, or delay the onset, of chronic diseases associated with ageing, such as Alzheimer’s and Parkinson’s diseases (Hoffman et al., 2017; DiLoreto & Murphy, 2015). *C. elegans* is an ideal model for the study of ageing as it has a relatively short lifespan of around 2–3 weeks under normal conditions (Corsi et al., 2015; Altun & Hall, 2009). Importantly, *C. elegans* lifespan has also been shown to be extended by single mutations in specific genes, such as components of insulin/IGF-1 like signalling pathways (van Heemst, 2010). The discovery of systems that act in ageing and their mechanisms of action in the model organism *Caenorhabditis elegans* may be applicable to the improvement and extension of heathy human lifespans.

Metabonomics, defined as “the quantitative measurement of the multiparametric metabolic response of living systems to pathophysiological stimuli or genetic modification” (Lindon et al., 2000; Everett et al., 2019), is a useful tool for investigating metabolic changes during ageing and age-related disease (Balashova et al., 2022). Among the factors that influence metabolism are diet, environment, disease, genetics and microbiome variation, resulting in a metabolic profile that reflects the health status of an individual (Everett et al., 2019). Metabonomics can also be used to investigate changes to the metabolism in model organisms such as *C. elegans*, and reveal relationships between genotype and phenotype that integrate across genomic and environmental factors, including the microbiome (Everett et al., 2019; Raamsdonk et al., 2001). The measurement of metabolites in biofluids or in tissue extracts of organisms such as *C. elegans* can be performed using NMR spectroscopy (Everett et al., 2019; Lindon et al., 2000), as in this study, or mass spectrometry (MS) (Scalbert et al., 2009; Watson, 2013).

Flavin-containing monooxygenases (FMOs) are NADPH-dependent enzymes, located in the membrane of the endoplasmic reticulum (ER), which catalyse the oxygenation of a wide variety of medicines and dietary-derived compounds (Krueger & Williams, 2005; Phillips & Shephard, 2020), detoxifying nitrogen- and sulphur-containing drugs and xenobiotics (Phillips & Shephard, 2017). Beyond their roles as xenobiotic-metabolising enzymes, however, FMOs are now known to be involved in various important endogenous functions in mammals. Human FMO1 was recently found to catalyse the conversion of hypotaurine to taurine, an amino acid critical for human health, utilising either NADPH or NADH as co-factor (Veeravalli et al., 2020). Also, host hepatic FMO3 is the primary FMO responsible for trimethylamine *N*-oxide (TMAO) production from trimethylamine. Genetic mutations reducing FMO3 activity result in trimethylaminuria (or fish-odour syndrome) in humans, caused by build-up of trimethylamine (Shephard et al., 2012; Yamazaki & Shimizu, 2007; Dolphin et al., 1997; Phillips & Shephard, 2020). FMOs are conserved across the eukaryotes and, notably, can be induced by multiple lifespan-extending interventions in mice: this poses the question over whether these enzymes might play a critical role in promoting health and longevity across phyla (Leiser et al., 2015). *Fmo5* knockout (KO) mice exhibited an age-related phenotype with lower body fat and weight, despite higher food intake, and lower blood glucose and cholesterol (Malagon et al., 2015). Changes in metabolism due to disruption of *Fmo5* indicated that metabolic ageing was slowed through pleiotropic effects (Malagon et al., 2015; Varshavi et al., 2018) and recently, a specific compound, 2,3-butanediol, was shown to be a microbiome-derived biomarker for *Fmo5* KO in mice. Moreover, 2,3-butanediol treatment prompted lower cholesterol and epididymal body fat in wild-type (WT) mice, recreating aspects of the phenotype of the *Fmo5* KO (Veeravalli et al., 2022).

Like mammals, the nematode *C. elegans* encodes five FMOs, again named FMO1-5 (Petalcorin et al., 2005). In the case of *Fmo-2*, overexpression (OE) in the *C. elegans* intestine was reported to increase worm lifespan through activation of hypoxia inducible factor (HIF)−1 (Uno & Nishida, 2016a; Leiser et al., 2015).

Correspondingly, *fmo-2* and also *fmo-4* transcription was upregulated by hypoxia (Shen et al., 2005). In more recent studies, oxidative stress (Goh et al., 2018) and infection with either *Pseudomonas aeruginosa* (PA14) (Dasgupta et al., 2020) or *Staphylococcus aureus* was found to induce *fmo-2*, with FMO-2 required for pathogen resistance (Wani et al., 2021). Recent work also supported a role for *fmo-2* in *C. elegans* innate immunity, as it’s transcription was strongly induced via NHR-49 and HLH-30 (Wani et al., 2021) in a pathogen-specific manner to impact infection survival. *C. elegans* FMO-4 was expressed prominently in hypodermis, duct and pore cells but was absent from excretory cells (Petalcorin et al., 2005). FMO-4 was hypothesised to possess an osmoregulatory role, promoting clearance of excess water that enters during periods of hypotonicity, potentially by synthesising an osmolyte that acts to establish an osmotic gradient from excretory cell to duct and pore cells (Hirani et al., 2016).

Previous observations of the role of mammalian FMO5 in ageing raised the possibility that modulation of all or any *C. elegans* FMOs may be a conserved mechanism for enhancing protein homeostasis and extending lifespan. The appropriate modulation of all FMOs might equally promote healthy ageing, improving health span, in mammals and people (Leiser et al., 2015). We recently demonstrated that knockout of *fmo-1*,* fmo-*3 and *fmo-*4 statistically significantly extended *C. elegans* lifespan relative to wild type (Said et al., 2024). Therefore, in this study, metabolic profiles were obtained and more detailed phenotypic analyses (i.e., development, behaviour, ageing and egg-laying and hatching) were systematically analysed for all *C. elegans fmo* KO lines, to further delineate or identify conserved roles for all *fmo’s* in development and ageing, as an aid to understanding the important endogenous roles emerging for FMO enzymes in mammalian metabolism.

## Results

### Phenotypic changes with FMO genetic variation

#### *fmo-2* KO, *fmo-3* KO and *fmo-4* KO delayed *C. elegans* development

Phenotypic differences were identified between WT and *fmo* mutant *C. elegans* worms in terms of development. The length of KO mutants of *fmo-2* (0.70 mm +/- 0.02; *p* < 0.003), *fmo-3* (0.58 mm +/- 0.015; *p* < 0.0001) and *fmo-4* (0.67 mm +/- 0.02; *p* < 0.0001) was significantly shorter at the beginning of day 3 post-hatching relative to WT (0.81 mm +/- 0.01), *fmo-1* KO (0.76 mm +/- 0.02; *p* > 0.05, not significant (ns)) and *fmo-5* KO worms (0.76 mm +/- 0.02; *p* > 0.05, ns) (Figure S1A). In addition, at the beginning of day 3, the plates of WT, *fmo-1* KO *fmo-2-*OE and *fmo-5* KO *C. elegans* contained the expected mix of eggs and mother worms, whereas plates of *fmo-2* KO, *fmo-3* KO and *fmo-4* KO contained mainly mother worms and were delayed in their development by approx. 5–6 h (Figure S1B). At the end of day 3, the worms of *fmo-2* KO, *fmo-3* KO and *fmo-4* KO mutants began to lay eggs, whereas on WT and *fmo-1* KO and *fmo-5* KO mutant plates, many eggs were visible (Figure S2).

#### Loss of *fmo-4* increased *C. elegans* chemical/osmotic sensitivity

A difference was observed in resistance to a strong oxidising agent in *fmo-*4 KO. During *C. elegans* egg preparation, the *fmo-4* KO strain resistance of its cuticle to bleach treatment (see Methods) was decreased compared with WT worms: the time required for bleach to disrupt ca. 95% of worms in *fmo-4* KO was 2.12 +/- 0.13 min; *p* < 0.0001), whereas the time required for WT *C. elegans* was 5.19 +/- 0.12 min (Figure S3A-C).

#### Loss of *fmo-4* in *C. elegans* was deleterious to embryo hatching

At day 3 post-hatching, a difference could be seen clearly in the number of adult *C. elegans* worms between WT and *fmo-4* KO. A mean (+/- SEM) of only 57 +/- 2% of *fmo-4* KO eggs hatched, compared with 95 +/- 2% of eggs hatching in WT (*p* < 0.0002, Figure S3D-G). Interestingly, *fmo-4* KO was the only *C. elegans* mutant that showed a large decrease in the number of adult worms in comparison to WT at day 3 post-hatching (data not shown).

#### *fmo-2* KO *C. elegans* swarming phenotype

**Fig. 1:**
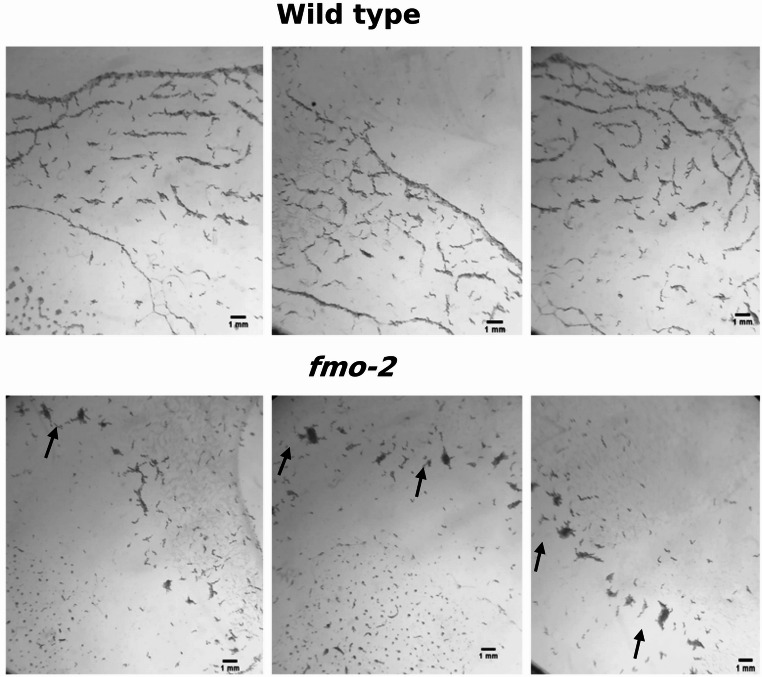
*fmo-2* KO *C. elegans* swarming phenotype. Groups of worms on the bacterial lawn (black arrows) were observed on *fmo*−2 KO plates versus WT worms spread across the plate

*fmo-2* KO worms feeding behaviour occurred in aggregates, with more coherent groups of worms swarming across a bacterial lawn (Ding et al., 2019) than seen for WT or the other four *fmo* knockout lines, *fmo-1*,*3*,*4* and *− 5*. This phenotype was evident from the first day of hatching, with *fmo-2* KO worms tending to move in groups from the centre to the edges of the bacterial lawn until they had consumed the bacteria (Fig. 1).

### Metabolic changes in *C. elegans* extracts with FMO genetic variation

#### Metabolic profiles of extracts of *Fmo* mutants at day 3 post-hatching

**Fig. 2:**
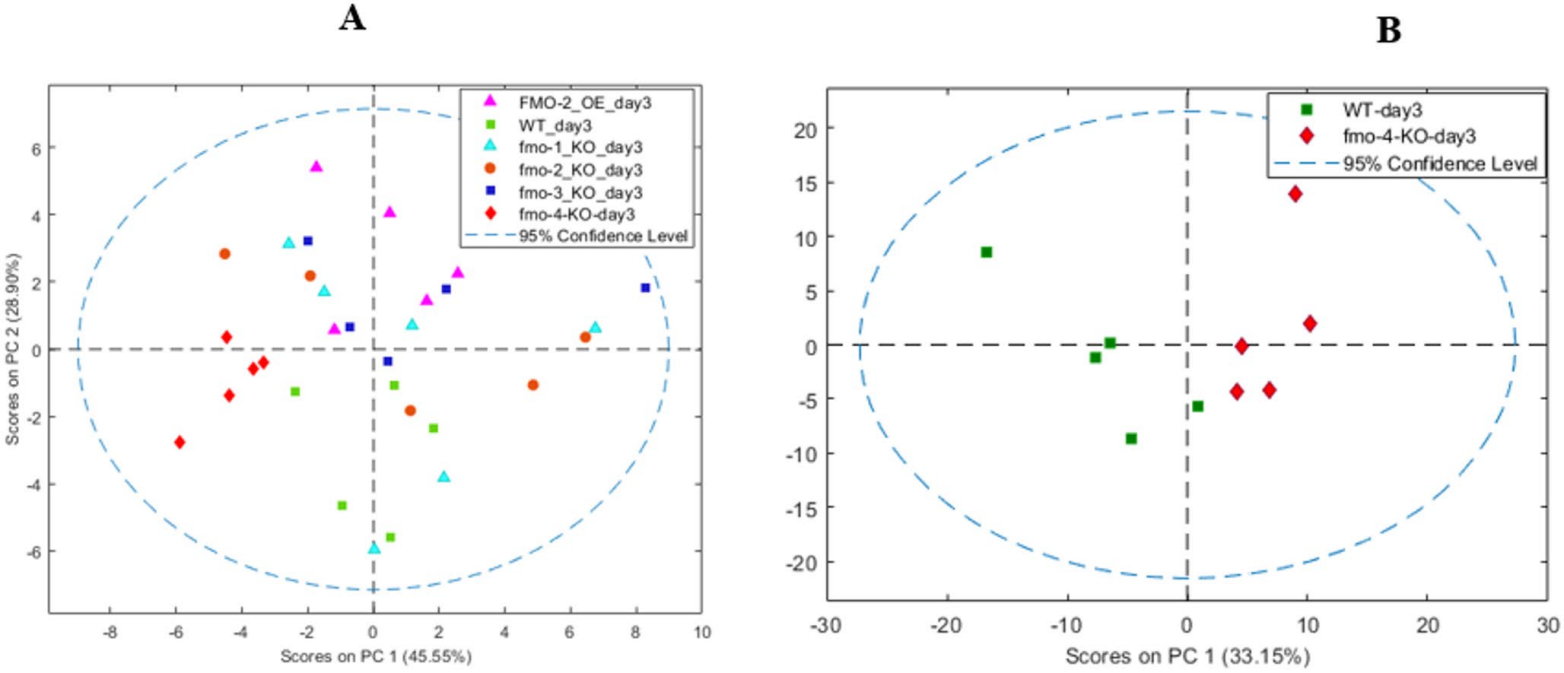
**A** PCA of data from 600 MHz ^1^H NMR spectra of metabolic extracts of WT and *fmo* mutant *C. elegans* worms at day 3 post-hatching. In the two-component model, PC1 explained 46% of the total variance and PC2 explained 29%. *N* = 5. **B** PCA of data from 600 MHz ^1^H NMR of metabolic extract of WT and *fmo-4* KO at day 3 post-hatching. In the two-component model, PC1 explained 33% of the total variance and PC2 explained 21%. *N* = 5

At day 3 post-hatching, unsupervised and unbiased principal components analysis (PCA) of all mutants showed no sample overlaps of *fmo-4* KO worm metabolite profiles on PC1 with those of WT (Figure 2A) and similarly for *fmo-4* KO alone vs. WT (Figure 2B, Figure S4). Moreover, *fmo-3* KO alone (Figure S5) had no sample overlaps with WT on PC1.

PCA of *fmo-2* KO vs. WT (Figure S6) showed a complete overlapping on PC1 and a partial overlap with WT on PC2, and the *fmo-2* OE extracts (Figure S7) versus WT showed no sample overlap on PC2. *fmo-1* KO (Figure S8) and *fmo-5* KO (Figure S9) were the only strains that had no PCA separation from WT in pairwise comparisons. Interestingly, at the earlier, embryo, life stage, PCA of NMR-based metabonomics of *fmo-4* KO and WT metabolite extracts showed no group separation (Figure S10). When the composition of the metabolome of each worm mutant was compared at day 3 post-hatching, using PCA and ANOVA, branched chain amino acids (BCAA; isoleucine, leucine and valine) were present at significantly higher levels in *fmo-2* KO, *fmo-4* KO and *fmo-2* OE strains than in WT (Table S1).

The levels of agmatine (a metabolite of arginine), phosphorylcholine and choline were decreased in *fmo-2* KO and *fmo-2* OE strains, whereas the levels of 5’-adenosine monophosphate (AMP), 5’-adenosine triphosphate (ATP) and 5’-uridine monophosphate (UMP) were decreased in *fmo-2* KO, *fmo-4* KO and *fmo-2* OE. In contrast, *fmo-4* KO showed increased threonine, phenylalanine and tyrosine. Finally, *fmo-3* KO showed an increased level of cystathionine, agmatine, lactate and threonine (Table S1).

At this day 3, post-hatching, timepoint, the metabolites discriminating *fmo-2* KO, *fmo-4* KO and *fmo-2* OE strains from WT shared similar pathways and included valine, leucine and isoleucine degradation (Figure S11, S12). Interestingly, *fmo-2* KO and *fmo-2* OE shared common pathway changes including phosphatidylcholine and phospholipid biosynthesis (Figure S11, S12) but differences between WT and the *fmo-2* OE line were observed in other pathways, such as mitochondrial beta-oxidation of short, medium and long chain fatty acids, ethanol metabolism, riboflavin metabolism and urea cycle, that did not occur in *fmo-2* KO extracts.

For *fmo-4* KO, the discriminating metabolites were also involved in phenylalanine and tyrosine metabolism, purine metabolism, glutamate metabolism and phenylacetate metabolism (Figure S12).

#### Metabolic profiles of extracts from *Fmo* mutants at day 6 post-hatching

**Fig. 3:**
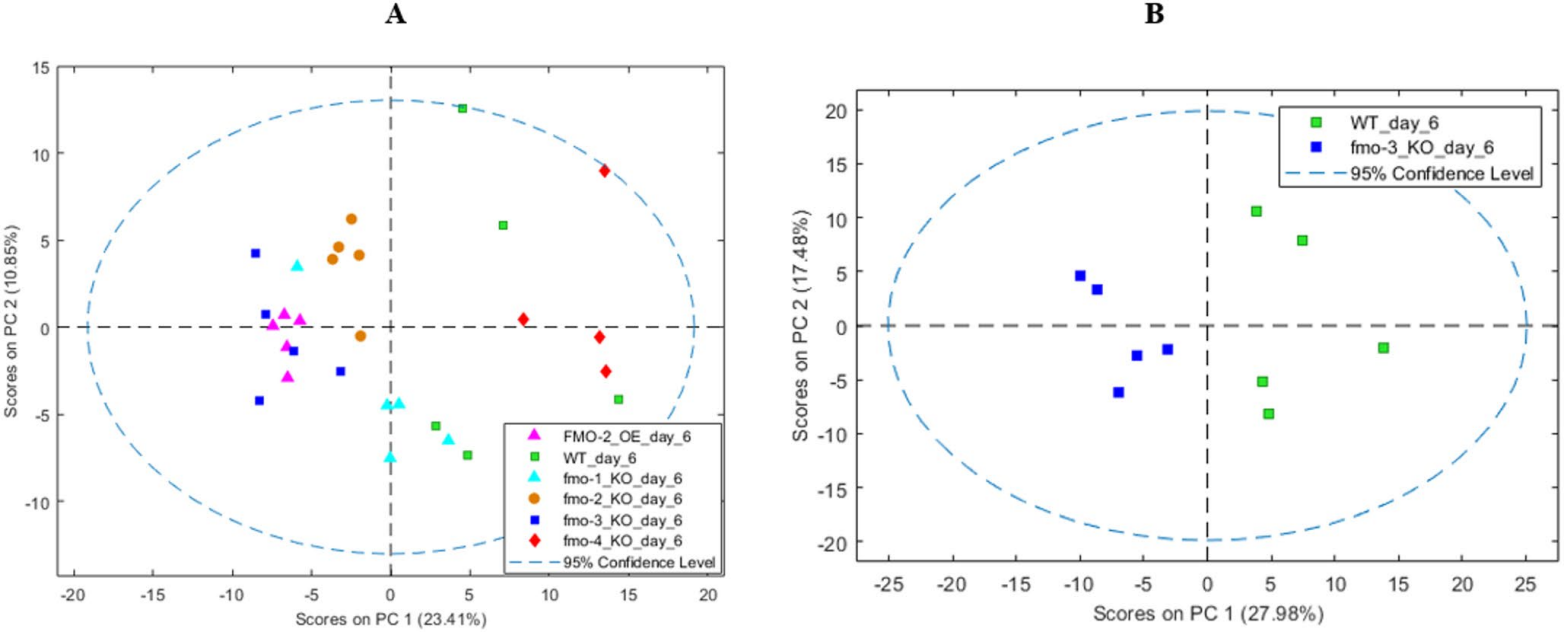
**A** PCA of data from 600 MHz ^1^H NMR of metabolic extracts of WT and *fmo* mutants at day 6 post-hatching. In the two-component model, PC1 explained 23% of the total variance and PC2 explained 11%. **B** PCA of data from 600 MHz ^1^H NMR of metabolic extract of WT and *fmo-3* KO at day 6 post-hatching. In the two-component model, PC1 explained 28% of the total variance and PC2 explained 18%

At day 6 post-hatching, PCA of NMR spectra of the extracts of WT and all *fmo* mutants combined showed no sample overlaps between the WT and *fmo-2* KO, *fmo-2* OE and *fmo-3* KO on PC1 (see Fig. 3A). *fmo-1* KO, in contrast, showed a degree of separation but with some overlap with WT. Conducting a PCA of WT separately versus each *fmo* mutant shows no sample overlaps with fmo*−3* KO (Fig. 3B; Figure S13), *fmo-2* KO (Figure S14) and *fmo-2* OE (Figure S15) on PC1. PCA of *fmo-4* KO (Figure S16) versus WT showed complete overlap on PC1 and some overlap on PC2, and the PCA of *fmo-1* KO (Figure S17) versus WT also showed some overlaps on PC1 and PC2. The metabolome of each mutant at day 6 post-hatching was once again evaluated for discriminating metabolites using PCA loadings and ANOVA (Table S2). The discriminating metabolites of each strain allowed enriched pathways to be constructed for each strain (Figures S18-19). In the two strains with increased lifespan (*fmo-3* KO and *fmo-2* OE) the discriminating metabolites were from pathways including the urea cycle and phenylacetate metabolism (Figure S19).

*fmo-2* OE also had discriminating metabolites involved in pyrimidine metabolism and nicotinate and nicotinamide metabolism (Figure S19) and those for *fmo-2* KO included betaine metabolism, and transfer of acyl groups into mitochondria (Figure S18).

#### Metabolic profiles of extracts of *Fmo* mutants at day 9 post-hatching

**Fig. 4:**
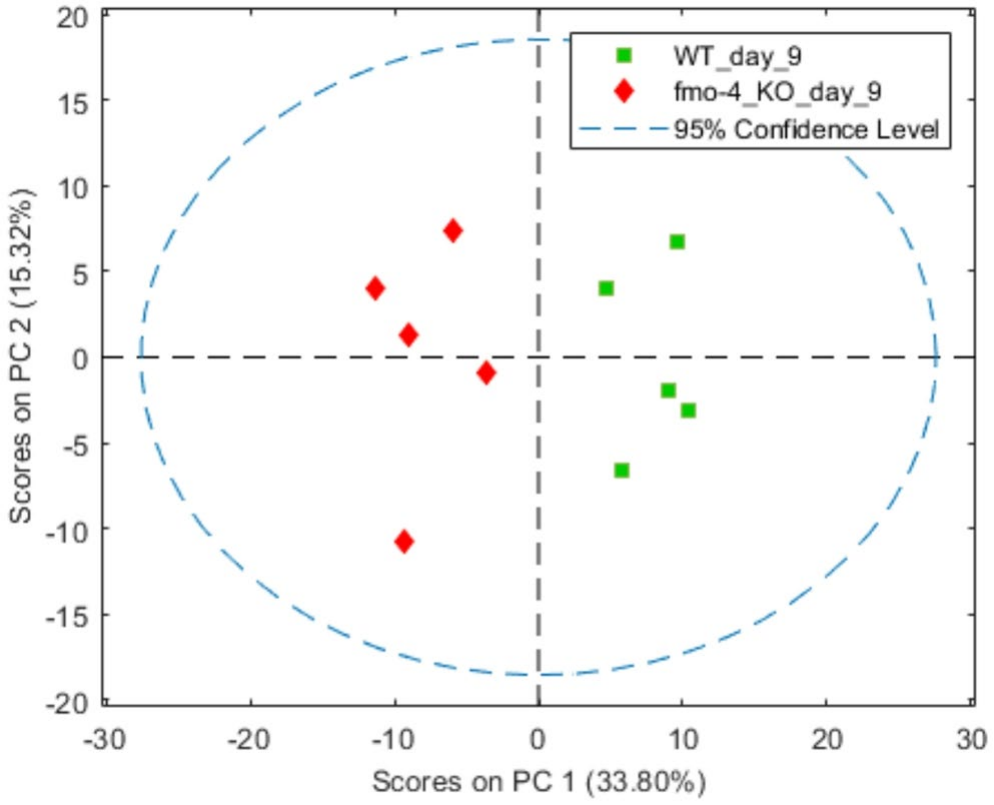
PCA of data from 600 MHz ^1^H NMR of metabolic extract of *C. elegans* WT and *fmo-4* KO at day 9 post-hatching. In the two-component model, PC1 explained 34% of the total variance and PC2 explained 15%

The metabonomics study extended to an additional timepoint for *fmo-4* KO vs. WT, at day 9 post-hatching, following its significant metabolic differences vs. WT at day 3, and because *fmo-4* KO had a significant lifespan extension over WT (Said et al., 2024). At day 9 post-hatching, PCA once again showed that *fmo-4* KO and WT had distinct metabolic profiles (Fig. 4, Figure S20) with increased levels of tryptophan, choline and phosphorylcholine, but decreased glutamine, asparagine, cystathionine, aspartate, agmatine, 5′-UMP, trehalose and 5’-guanosine monophosphate (GMP) in *fmo-4* KO (Table S3). The discriminating metabolites of *fmo-4* KO at day 9 post-hatching were involved in several pathways including aspartate metabolism, valine, leucine and isoleucine metabolism, phosphatidylcholine metabolism and betaine metabolism (S21).

#### Metabolic profile changes as a result of ageing in WT and *Fmo* mutant worms

Having characterised WT and *fmo-4* KO worms at three different stages (day 3, 6 and 9 post-hatching), PCA of NMR-based metabolite extract profiles determined the metabolic trajectories of both strains over time. Both strains followed a similar metabolic trajectory from day 3 to day 6 post-hatching, with both groups moving to lower PC1 values and becoming more diffuse and overlapped (Figure S22). Notably, from day 6 to day 9, the WT spectral profiles did not move whereas the *fmo-4* KO worm metabolic profiles moved to lower PC1 and PC2 values and became distinct again from the WT (Figure S22).

The metabolic changes that occurred with ageing were identified by comparing ^1^H NMR metabolic profiles of WT and mutant worms at day 3 post-hatching relative to those at day 6 and day 9 post-hatching stages (Table S4-S11). The levels of alanine, lactate, asparagine, agmatine, succinate, cystathionine, histidine, 5′-AMP, 5′-ATP and 5′-UMP were decreased in day 6 post hatching WT worms whereas D-glucose levels were increased at days 6 and 9 compared with day 3 post-hatching (Table S12).

Most of the *fmo* mutants exhibited patterns of ageing progression that largely or partially overlapped with that of the WT worms, with changes particularly in for glucose, alanine, lactate, succinate and agmatine levels as the worms aged from day 3 to day 6 post-hatching (Table S12). The most notable differences in the long-lived lines were the increased glutamine, asparagine, aspartate and glutamate levels in *fmo-2* OE and increased tryptophan in *fmo-4* KO, *fmo-3* KO and *fmo-2* OE strains.

Differences were seen between *fmo-4* KO and WT lines at day 9 post-hatching (see Figure S20-21), with glutamine, glutamate, leucine, citrate and 5′-GMP decreased, and the levels of formate, tryptophan, choline, phosphoryl choline, ethanol and methanol, increased in the day-9, *fmo-4* KO strain (Table S12).

## Discussion

### Phenotypic changes due to genetic variation

Our previous study showed that the loss of *fmo-1*, *fmo-3* and *fmo-4* and the overexpression of *fmo-2* increased longevity compared with WT *C. elegans* worms (Said et al., 2024). In this study, we showed that *fmo-4* KO, *fmo-2* KO and *fmo-3* KO worms exhibited a delayed developmental phenotype, reaching the adult stage about 4–5 h later than WT, timed from the beginning of egg production. *Fmo-1* KO and *fmo-2* OE mutants did not show the same developmental delay. (Table 1; Figure S1). These results indicated that FMO enzymes have a role in *C. elegans* development and in extending *C. elegans* lifespan. Although another recent study reported that *fmo-4* KO had no effect on *C. elegans* lifespan (Tuckowski et al., 2025), feeding levels were higher in the latter study, a difference in experimental conditions that could affect this model, which is highly sensitive to environment and diet (Ezcurra et al. 2011).

The *fmo-2* KO was the only *C. elegans* mutant studied here that showed a distinctive behaviour phenotype, of swarming or aggregation (Table 1; Fig. 1). Swarming is one of the most complex social behaviours exhibited by *C. elegans* (Avery et al., 2021). It was reported that starvation in larvae (L1) induced swarming (Artyukhin et al., 2015). *C. elegans* was reported to exhibit a strong behavioural preference for 5–12% oxygen, avoiding lower or higher levels of oxygen, and a link with both swarming and starvation is found in the observation that social feeding occurred only when oxygen exceeded the preferred level (Gray et al., 2004). Mutation in the *npr-1* gene, which encodes a predicted G protein-coupled receptor similar to neuropeptide Y receptors, causes a solitary strain to take on social behaviour (de Bono & Bargmann, 1998), suggesting the inclusion of Npr1 in further experiments to explain swarming behaviour upon loss of *fmo-2*.

Phenotypic analysis supports an important role in *C. elegans* for *fmo-4* as its loss both increased sensitivity to bleach treatment (Figure S3) and increased embryo lethality relative to WT. The decreased rate of egg hatching was not a result of decreased egg formation rate (Table 1; Figure S3). The increased sensitivity to bleach treatment agrees with a reported osmoregulatory role for *fmo-4*, loss of which affects clearance of excess water during hypotonicity (Hirani et al., 2016). A role for *fmo-4* is plausible in the synthesis of an osmolyte that acts to establish an osmotic gradient from excretory cell to duct and pore cells (Hirani et al., 2016). Alternatively, loss of *fmo-4* could affect composition of the cuticle of the worm, which provides the first line of defence against chemical and microbial stressors (Page & Johnstone, 2007).

There may be other, redundant functions for *fmo-4* that, at least partially, overlap with *fmo-2*, since inactivation of *fmo-4* led to up-regulation of *fmo-2* by ca. 15- to 30-fold (Said et al., 2024). Shared *fmo-2* functions would therefore be interesting regarding the *fmo-4* KO’s differences in egg hatching and worm development (Table 1).

This study uncovered several new potential endogenous effects of *C. elegans fmo*s. Table 1 shows the different phenotypes associated with different *fmo* mutant strains.

**Table 1:** Summary of phenotypes of *Fmo* mutant *C. elegans* worms

Strain	Phenotypes			
	Lifespan (Said et al., 2024)	Development	Egg hatching	Distinctive social behaviour
*fmo-1* KO	Extended lifespan	No effect	No difference observed	No effect
*fmo-****2*** KO	No effect	Delayed development	No difference observed	Aggregation and grouped feeding behaviour
*fmo-3* KO	Extended lifespan	Delayed development	No difference observed	No effect
*fmo-4* KO	Extended lifespan	Delayed development	Decreased hatching rate	No effect
*fmo-5* KO	No effect	No effect	No difference observed	No effect
*fmo-2* OE	Extend lifespan	No effect	No difference observed	No effect

### Metabolic profile changes in adult *C. elegans* worms due to genetic variation

The metabolome of each strain was analysed at day 3 post-hatching to compare the effect of each *fmo* mutation on the adult stage worm composition. Multivariate analysis (PCA) revealed that all *fmo* mutant strains possessed a distinct metabolic profile compared with WT, except for *fmo-1* KO, which had a similar although more diffuse metabolome (Figure S8).

Higher levels of BCAAs were observed in *fmo-2* KO, *fmo-4* KO and *fmo-2* OE strains compared to WT (day 3; by PCA and ANOVA, Table S1). Like other animals, *C. elegans* cannot synthesise BCAAs (Payne & Loomis, 2006), and so any difference in their relative concentrations must be due to a change in either protein turnover or BCAA catabolism (Brosnan & Brosnan, 2006). Down-regulation of the branched-chain α-ketoacid dehydrogenase complex was hypothesised to be responsible for increased BCAA pool sizes in a mutant in a developmental arrest gene, *daf-2* (Fuchs et al., 2010) which was linked with longevity, and affects fertility and embryonic development. The increased day-3 BCAAs levels in the *fmo-4* KO, which had effects in all these aspects of the *C elegans* life cycle, may be caused by the same mechanism.

*fmo-4* KO also showed increased levels of threonine, phenylalanine and tyrosine at day 3 post-hatching compared with WT. These changes of amino acid level in the *fmo* mutants could also result from a role in development for *fmo-4* because these amino acids are the building blocks of proteins formed during growth and development of an organism (Edwards et al., 2015).

Interestingly, the levels of agmatine, phosphorylcholine (PCho) and choline were decreased in both KO and OE strains of *fmo-2*. PCho is produced from choline phosphorylation by choline kinase. Meanwhile, endoplasmic reticulum (ER) stress activated choline kinase (CKB-2) expression, which was linked with ageing, and low phosphocholine (PCho) was correlated with high life expectancy (Pontoizeau et al., 2014). The decreased level of PCho in these *fmo-2* mutant worms tallies with this and could therefore be related to effects on or from CKB-2.

### Metabolic profile changes in adult *C. elegans* worms due to ageing

If the metabolome is causally linked to lifespan extension, then the metabolomes of *fmo-1* KO, *fmo-3* KO, *fmo-4* KO and *fmo-2* OE mutants should be different from that of WT at some, or even all, stages of the *C. elegans* life cycle.

Multivariate analysis by PCA showed a distinct metabolic profile compared with WT for the *fmo-4* KO *C. elegans* mutant at days 3 and 9 but not at day 6 post-hatching. The *fmo-1* KO was the only lifespan-extended strain that did not show any metabolic separation at day 3 or 6 post-hatching (Figures S8 and S17). The lack of effect of removing *fmo-1* on ^1^H NMR-detectable *C. elegans* metabolite profiles suggests that lifespan extension may not always be correlated with patent metabolite profiles changes. The number of significantly different metabolites between *fmo-2* KO, *fmo-2* OE and *fmo-3* KO relative to WT, however, increased with ageing (Table S1 & S2).

There were no significant differences in the BCAA levels at day 6 post-hatching between WT and any *fmo* mutants (Table S2) but BCAA levels were down-regulated in *fmo-4* KO at day 9 post-hatching (Table S3). The same was also seen in two other long-lived strains, namely *eat-2*(*ad465*) and *slcf-1*(*tm2258*), both of which had decreased leucine vs. WT in 7-day-old adult worms (Pontoizeau et al., 2014). However, the metabolomes of both *glp-1* KO and the long-lived *daf-2* mutant, mentioned above, showed elevated levels of BCAAs at 10 days’ of age (Fuchs et al., 2010); (Wan et al., 2017).

The abundance of PCho and choline pathway activation with age (Pontoizeau et al., 2014) means that the increased level of PCho in 9-day, *fmo-4* KO worms could be due to CKB-2 activation, perhaps to compensate for stress from ageing. This contrasts, however, with a previous study of other long-lived mutants which concluded that low PCho levels correlated with high life expectancy in *C. elegans* (Pontoizeau et al., 2014) (Fuchs et al., 2010). The results for *fmo-4* KO at day 9 post-hatching, with increased levels of PCho, choline and trimethylglycine, are consistent with reports of the WT dauer metabolome which shows elevated levels of phosphoserine, hydroxyproline and choline compounds (Fuchs et al., 2010). These changes were also correlated for long lived mutants in this study and also for dauer stage nematodes, grown in the same conditions (Fuchs et al., 2010).

The long-lived mutants, *fmo-3* KO and *fmo-2* OE at day 6 and *fmo-4* KO at day 9 post-hatching, showed higher levels of tryptophan relative to WT, seen previously in dauer stage and long-lived mutants *daf-2* and *ife-2* (Fuchs et al., 2010). In *C. elegans*, blocking tryptophan catabolism may extend lifespan via regulation of proteotoxicity (accumulation of damaged or misfolded proteins) (van der Goot et al., 2012) whereas in human serum, tryptophan levels decreased with ageing (Yu et al., 2012) and toxic tryptophan catabolites increased (Ramos-Chávez et al., 2018). An increase in tryptophan catabolism with ageing may result from increased levels of the enzyme indoleamine-2, 3-dioxygenase (IDO) whereas depletion of tryptophan 2, 3-dioxygenase (*tdo-2*) increased tryptophan levels (Ramos-Chávez et al., 2018). Literature reports (Edwards et al., 2008;^−^Bennett & Kaeberlein, 2014; Bennett et al., 2014), support a role for the increased tryptophan levels seen in *fmo-3* KO, *fmo-4* KO and *fmo-2* OE mutants in extending lifespan in these strains.

Although there were some differences between the results of the present work and previous ageing studies, metabolomes are sensitive to precise experimental conditions and genetic changes, the exact life-stage targeted, and to extraction and analysis methods. Furthermore, using FUdR resulted in genotype-specific effects (in *daf-2* mutant vs. WT nematodes) on levels of eight specific metabolites (Davies et al., 2012), but collecting sufficient biomass for metabolite analysis would not be possible without the use of FUdR, or a similar intervention, to synchronise cultures (Davies et al., 2012).Therefore, we developed a gravity worm filtering protocol (Said et al., manuscript in preparation) removing the need for use of FUdR. It is likely therefore that there would be some variation between the results in this study compared with those studies that made use of chemically synchronised samples.

### A proposed mechanism of lifespan extension for *Fmo* variants

One of the major functions of HLH-30 is to regulate autophagy and promote longevity in *C. elegans* (Lapierre et al., 2013; O’Rourke & Ruvkun, 2013). *fmo-2* is a target of HLH-30, and was induced both by hypoxia and starvation (complete bacterial food source removal) and induction by starvation was dependent on HLH-30 (Leiser et al., 2015).

The life-extending effects of hypoxia in *C. elegans* begin in neurons with HIF-1-upregulated transcription and increased serotonergic signalling (Leiser et al., 2015). These effects increased production of FMO-2 in the intestine, and increased longevity, whereas knocking out *fmo-2* did not affect *C. elegans* lifespan (Leiser et al., 2015). The latter findings (Leiser et al., 2015) were similar to those in the present study.

A hypothesis whereby increased levels of formate confer stress resistance and lifespan extension under metabolically stressful conditions, such as hypoxia or DR (Choi et al., 2023), could explain increased tryptophan and formate in *fmo-4* KO at day 9 post-hatching, and could link *C. elegans* lifespan extension in *fmo-4* KO with that in the *fmo-2* OE and *fmo-3* KO mutants (Choi et al., 2023).

Interestingly, *fmo-1* KO was a long-lived mutant that did not show elevated tryptophan with ageing. Thus, we propose that *fmo-1* KO extends worm lifespan via a different mechanism. This is also supported by the previous study with the same lines, in which *fmo-2* transcription was upregulated 15- to 30-fold compared with WT upon loss of *fmo-4*, whereas loss of *fmo-1* up-regulated *fmo-2* by only approx. threefold (Said et al., 2024). In contrast, long-lived *tald-1* mutant *C. elegans* had 30–40-fold increased *fmo-2* transcription relative to WT (Bennett et al., 2017). Thus, the up-regulation of *fmo-2* upon loss of *fmo-4* could be the cause of lifespan extension in the *fmo-4* KO strain.

**Fig. 5:**
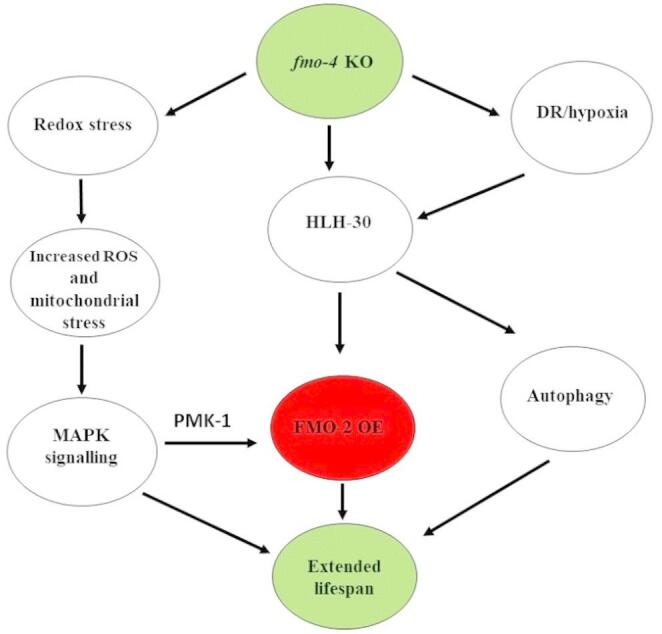
A model for *fmo-4* KO-mediated longevity

As the overexpression of *fmo-2* was reported to extend *C. elegans* lifespan in four different conditions including hypoxia, DR (Leiser et al., 2015), MAPK and HLH-30 activation, (Bennett et al., 2017), an important future experiment would be to determine which environmental condition or transcription factor is responsible for *fmo-2* up-regulation in *fmo-4* KO. The proposed, linked mechanisms of action in *fmo-4* KO resulting in extended *C. elegans* lifespan are shown in Fig. 5.

## Conclusion

This study has shown that there are interlinked but non-redundant roles for *C. elegans fmo*s, and revealed phenotypic differences between *C. elegans fmo* mutants. Loss of *fmo-4* significantly affected on egg hatching and sensitivity to bleach, but, for example, the *fmo-2* KO strain showed distinct swarming and aggregation behaviour. The metabolome of the long-lived *fmo-3* KO, *fmo-4* KO and *fmo-2* OE lines displayed higher tryptophan levels during ageing. *fmo-3* KO and *fmo-4* KO, but not *fmo-1* KO, may therefore extend the *C. elegans* lifespan via the same mechanism as *fmo-2* OE. Because *fmo-4* KO showed significant upregulation of *fmo-2* over WT levels, it was hypothesised that overexpression of *fmo-2* in *fmo-4* KO was responsible for *fmo-4* lifespan extension. Further experiments are needed, including identifying transcription factor involvement, to confirm whether *fmo-4* KO and *fmo-2* OE do indeed act by the same mechanism.

## Materials and methods

### *C. elegans* strains and maintenance

Animals were cultures at 20 °C and maintained on OP50 seeded NGM plates. *C. elegans* strains were purchased from the *Caenorhabditis* Genetic Center (CGC, Minnesota, USA). *C. elegans* strains used in this study: Bristol (N2) strain as the wild-type strain, *fmo-1* KO (RB671 [*fmo-1*(*ok405*) *IV*]), *fmo-2* KO (VC1668 [*fmo-2*(*ok2147*) *IV*]), *fmo-3* KO (RB686[*fmo-3*(ok354) III]), *fmo-4* KO (RB562 [*fmo-4*(*ok294*) *V*]), *fmo-5* KO (tm2438) and *fmo-2* OE (KAE10 [*seaSi40 I; unc-119(ed3) III*]).

### Worm length measurement

Synchronised cultures of WT and *fmo* mutants (*fmo-1* KO, *fmo-2* KO, *fmo-3* KO and *fmo-4* KO) were obtained using our developed egg preparation protocol. Egg of each strains were transferred to a new seeded NGM plate and left for 3 days until they reached the adult stage. 15–20 worms of each strain were immobilised using 40 µl of 1mM tetramisole hydrochloride (levamisole; Sigma). Images were taken using an M80 stereomicroscope (Leica, Wetzlar, Germany). Worm length was measured using Image J (Schindelin et al., 2012). The analysis was done using One-way ANOVA. Worm length of WT and *fmo-5* KO was also measured using an AI software (*C. elegans* length measurement; Rapid Biolabs).

### Bleach time test (cuticle sensitivity test)

0.2 ml worm pellet of each strain were re-suspended in 7 ml M9 buffer, and 2 ml of 1 M NaOH and 3 ml of 7.5% sodium hypochlorite (bleach) were added to each sample. The time for 95–100% of worms to be disrupted of each strain was counted. Each experiment consisted of three biological replicates and the analysis was done using student t-test.

### Egg hatching rate

For each strain, ~ 100–150 freshly laid eggs were transferred to each of three NGM plates and incubated at 20 °C for three days. Eggs were obtained either by standard egg preparation or by direct picking from source plates. After incubation, all worms that hatched (larvae and adults) were counted on each plate. The number of unhatched embryos was calculated by subtracting the number of hatched worms from the initial number of transferred eggs. Hatching percentage was then determined as (number of hatched worms ÷ total eggs transferred) × 100. The mean and SEM of the three repeats for each strain were calculated, and plotted using Graphpad Prism 6.0 software (La Jolla, California, USA). In this way, embryos that failed to hatch, including developmentally arrested or non-viable embryos, were included in the scoring. The data were analysed using student t-test. Images of each plate was taken to double check the number of adult worms and to determine the visual differences between strains.

### Egg laying rate

Normally this assay was done by letting number of L4/adult worms to lay eggs for 12 h and repetitive transfer to new plates every 12 h and let eggs laid on each plate to hatch, two days later, number of worms per each plate was counted. This normal assay is not appropriate to the strains with reduced egg hatching rate as the count of hatched eggs will not be indicative for the total number of eggs laid. Modified egg laying rate was developed to overcome this problem, this assay aimed to count eggs instead of counting worms post hatching. 3–5 adult (1st day of adulthood) worms were transferred to 35 mm NGM plates seeded with OP50. All plates were left for 12 h. Number of eggs and hatched larvae were counted and recorded. The advantages of the modified assay was being suitable for the stains with reduced egg hatching rate, it was a combination of egg hatching and egg laying rate as results could be divided into number of laid eggs in 12 h and the rate of eggs hatched could be calculated from the ratio of hatched worms to the total number of laid eggs.

### Maintaining and growing *C. elegans* to the required stage

*C. elegans* WT and different *fmo* mutants (*fmo-1* KO, *fmo-2* KO, *fmo-3* KO, *fmo-4* KO, *fmo-5* KO and *fmo-2* OE) were tested metabolically at day 3 and day 6 post-hatching, in addition WT and *fmo-4* KO were also tested at day 9 post-hatching. Different strains were maintained and the synchronised cultures were obtained by using the developed egg preparation protocol. 100 µl egg pellet was transferred to each OP50 seeded NGM plate, hatched larvae were checked under the microscope daily for growth. For each strain, 5 different biological repeats were obtained at each tested stage. Worms were combined from three individual 90 mm NGM plates to make a single replicate. For day 3 post-hatching stage, once they reach the adulthood stage (defined as the point where there were eggs seen on the plates but no new-generation worms had hatched), they were ready for metabolite extraction. The adult worms of each strain were collected in 15 ml Falcon tubes using M9 buffer, all tubes were spun at 1,300 *g* for 1 min. The supernatant was discarded, worm pellets were washed three times with 10 ml M9 buffer to get rid of the *E. coli*. Samples were snap frozen and stored at −80 °C until metabolite extraction.

For the day 6 and 9 post-hatching stages, worms of each strain after they reached the adult stage (day 3 post-hatching) were washed and filtered from the larvae worms (new progeny) daily using our developed gravity worm filter protocol (Said et al., manuscript in preparation).

in preparation). Mother worms were transferred to new seeded plates to supply the worms with OP50. At each stage, worms were collected and harvested using the same method as day 3 stage. Metabolite extraction of *C. elegans* is critical, and required sufficient worms to give a 0.3–0.5 ml pellet of synchronised adult worms, so these steps were repeated several times to obtain the ideal mass of the required worms.

### Metabolite extraction and sample Preparation for NMR analysis

Metabolite extraction from different *C. elegans* strains was performed using methanol and worms were disrupted using zirconium beads (Sigma, Dorset, UK) for 5 min in TissueLyser II (Qiagen) at 30 Hz. The metabolite extracts of all *C. elegans* strains were dried overnight in a RapidVap Vertex Evaporator (LABCONCO, UK) before NMR analysis. Dried extracts were vortexed and resuspended with 240 µl of phosphate buffer (pH. 7.4; 0.93 g NaH_2_PO_4_ (Fisher), 1.04 g K_2_HPO_4_(Fisher), 0.86 mg TSP (Sigma) and 5.85 mg sodium azide (Fisher), all dissolved in 10 ml D_2_O (Sigma)). Eppendorf tubes were centrifuged for 10 min at 12,000 *g* at 4 °C. 200 µl of each extract was transferred to new 3 mm diameter NMR tubes (SampleJet Tube 3.0 × 103.5 mm) by using eVol XR electronic syringe (SGE-Analytical Science, UK).

### Nuclear magnetic resonance spectroscopy

All sample were analysed at 300.0 K by 600 MHz ^1^H NMR (Avance III HD spectrometer, Bruker Biospin, Karlsruhe, Germany) using a cooled Bruker Sample Jet to store the samples prior to pre-heating and then insertion in the magnet. 1D ^1^H NMR spectra were acquired with the Bruker 1D NOESY water suppression pulse sequence noesygppr1d. 128 scans were accumulated into 32 K data points with a sweep width of ca. 20 ppm (12,019 Hz) and a relaxation delay of 4.0 s. The ^1^H NMR free induction decays were zero-filled to 128 K data points and apodised with a line broadening of 0.3 Hz, prior to Fourier transformation, manual phasing and baseline correction where necessary using MNova version 12.0.1.20560 (Mestrelab, 2018). Various 2D ^1^H NMR experiments were acquired as previously described (Veeravalli et al., 2022).

### Metabolite identification

Metabolites were identified by three complementary methods: (I) comparison with reference spectra from the human metabolome database (HMDB; http://www.hmdb.ca/; Wishart et al., 2007) and the biological magnetic resonance data bank (BMRB; http://www.bmrb.wisc.edu/metabolomics/); (II) analysis of previously published data; and (III) the interpretation of a series of two-dimensional (2D) spectra such as ^1^H COSY, JRES and HSQC, using published methods (Dona et al., 2016; Garcia-Perez et al., 2020).

### Multivariate statistical analyses

Regions of the ^1^H NMR spectra without signals (upfield of 0.5 ppm and downfield of 9.5 ppm) and the region containing residual water signals at ca. 4.8 ppm were removed. The spectra were normalised to a total signal area of 100 and then superimposed on one another by stacking in MNova (Mestrelab, 2018). The spectra were saved as a csv file (transposed comma separated variable) and then imported into Matlab 2018b (Mathworks, UK) for processing as a cellular array.

The data were carefully aligned using the icoshift algorithm (Tomasi et al., 2011), the relevant sample metadata (sample name, sample genotype, sample age) were imported and the spectra were bucketed into segments typically of 0.04 ppm spectral width. Further statistical analysis was performed in PLS-Toolbox 9.3 (Eigenvector USA) using standard methods such as principal components analysis (PCA), partial least squares discriminant analysis (PLS-DA). FDR Control used the method of Benjamini and Hochburg (Benjamini, 2010).

## Supplementary Information

Below is the link to the electronic supplementary material.

Supplementary material 1 (PDF 8327 kb)

## Data Availability

The original NMR spectroscopy data from this study has been deposited at MetaboLights (Yurekten et al., 2007): https://www.ebi.ac.uk/metabolights/at deposit MTBLS11908.
