# Supplementary material for: Phenotypic and metabonomics studies of FMOs in C. elegans and their roles in lifespan extension
Source: Metabolomics. 2025 Nov 15;21(6):170. doi: 10.1007/s11306-025-02367-4 (PMC12619732; doi:10.1007/s11306-025-02367-4)
Supplement: Supplementary file 1 — Supplementary material 1 (PDF 8327 kb) [file 11306_2025_2367_MOESM1_ESM.pdf]

Supplementary Information:

Phenotypic and metabonomics studies of FMOs in *C. elegans* and their roles in lifespan extension

Mohamed Said[1,#], Rafael Freire[1], Filipe Cabreiro[2,3], Jose Ivan Serrano-Contreras[4], Elinor P Thompson*[1], Jeremy Everett*[1]

1. Faculty of Engineering and Science, University of Greenwich, Chatham Maritime, Kent ME4 4TB

2. Institute of Clinical Sciences, Imperial College London W12 0NN, UK

3 - Cologne Excellence Cluster for Cellular Stress Responses in Ageing-Associated Diseases (CECAD), University of Cologne, Joseph Stelzmann Strasse 26, 50931 Cologne, Germany

4- Faculty of Medicine, Department of Metabolism, Digestion and Reproduction, Commonwealth Building, Hammersmith Campus, Imperial College, Du Cane Road, London, W12 0NN, UK

#- Current address: Faculty of Pharmacy, MSA University, 6th October City, Giza, Egypt

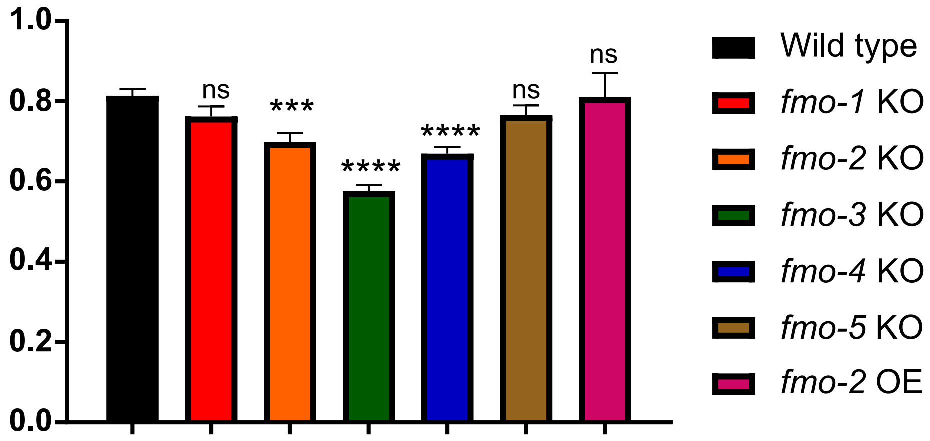

Figure S1A. *fmo-2* KO, *fmo-3* KO and *fmo-4* KO delayed *C. elegans* development relative to WT. Mean length (± SEM) at day 3-post hatching calculated from 15 worms per *fmo* line. One-way ANOVA: ***, $p < 0.001$, **** $p < 0.0001$, ns = not statistically significant.

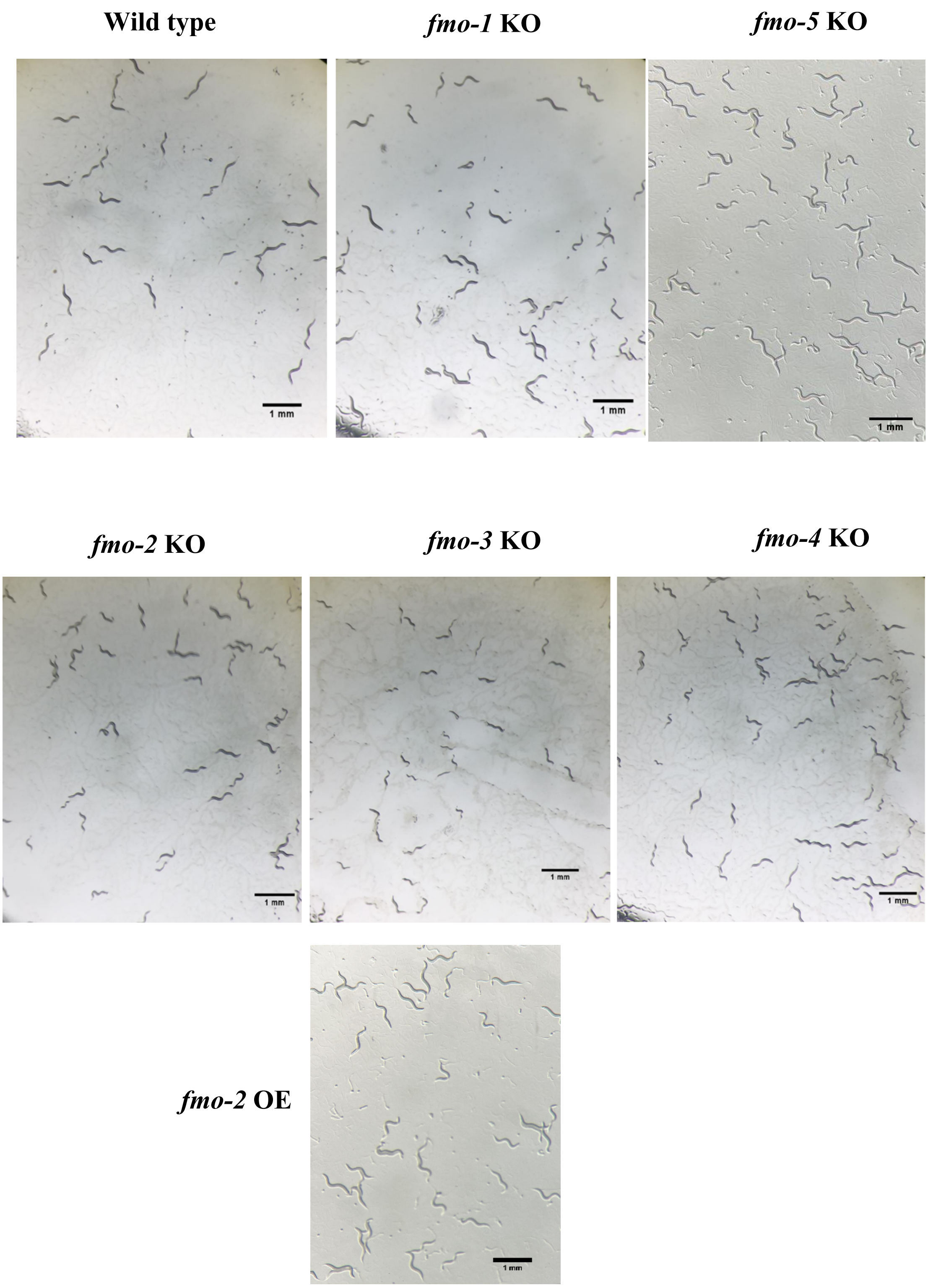

Figure S1B. Images of wild type and *fmo* mutant *C. elegans* worms at the beginning of day 3 post-hatching. *fmo-2* KO and *fmo-4* KO began to have a few eggs, *fmo-3* KO plates contained only young adults whereas the wild type, *fmo-1* KO, *fmo-2* OE and *fmo-5* KO plates contained mother worms and eggs.

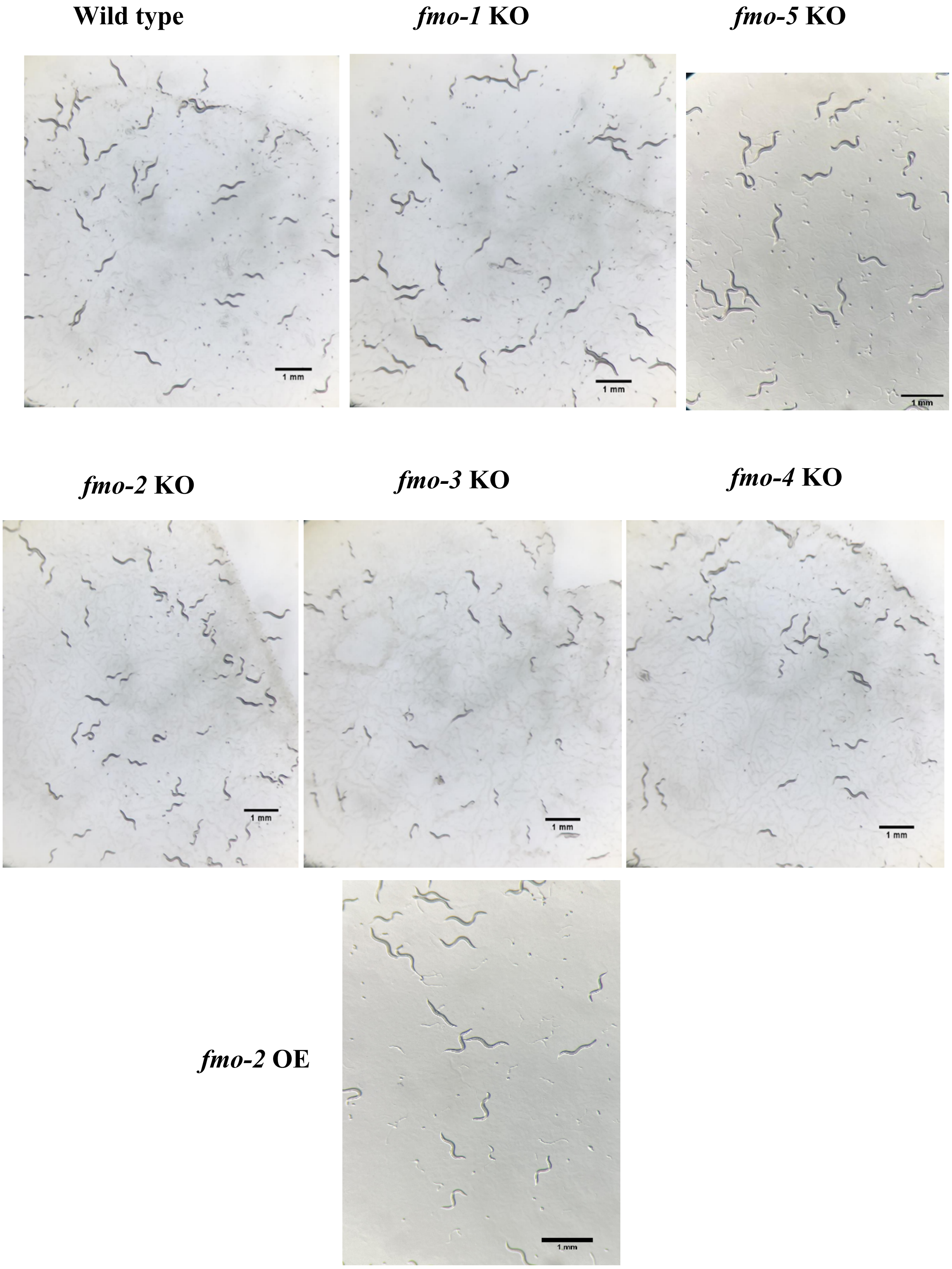

Figure S2. Images of wild type and *fmo* mutant *C. elegans* worms at the end of day 3 post-hatching. In *fmo-2* KO and *fmo-4* KO plates the number of eggs began to increase, *fmo-3* KO plates began to have few eggs whereas the wild type, *fmo-1* KO, *fmo-2* OE and *fmo-5* KO plates contained mother worms and many eggs.

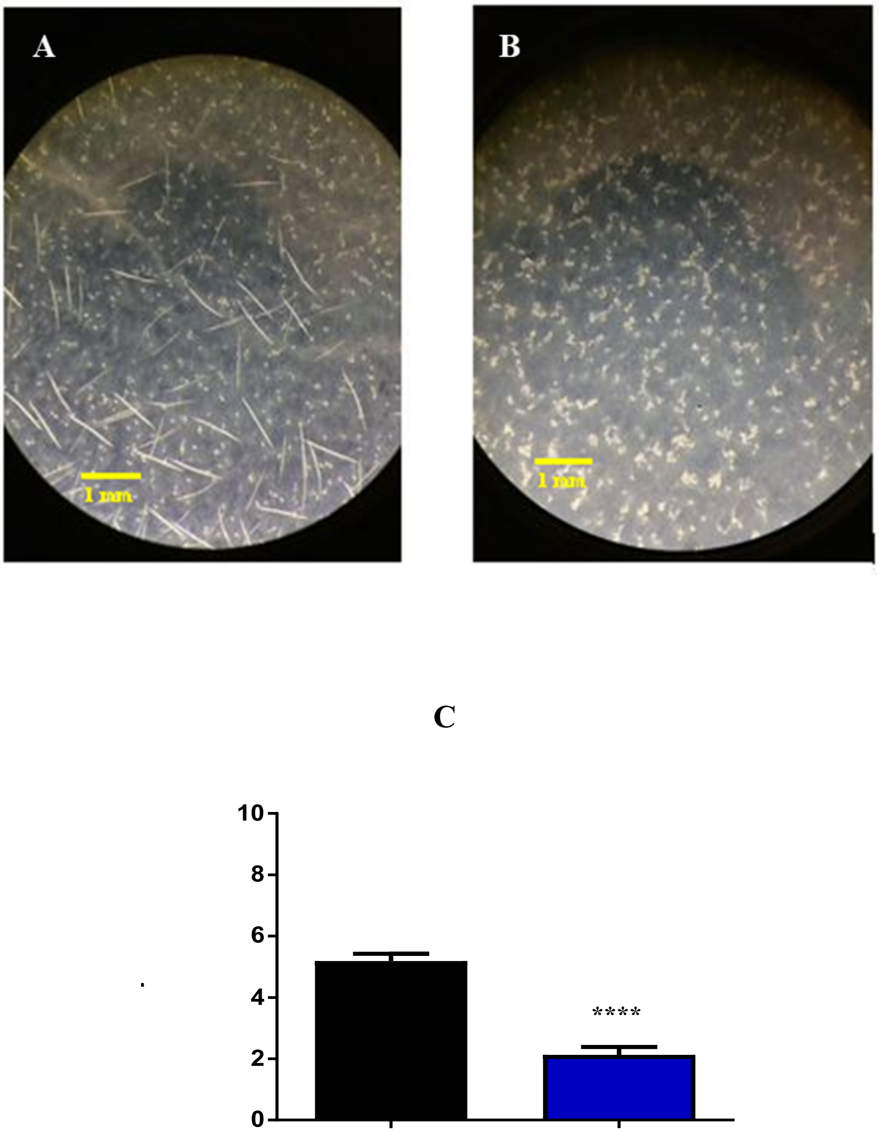

Figure S3. Loss of *fmo-4* increased sensitivity to bleach in *C. elegans*. **A**, Adult WT in bleach solution after 4 min. **B,** Adult *fmo-4* KO in bleach solution after 2 min. **C,** Mean time to disruption ± SEM (t test, $p < 0.0001$; $n = 3$).

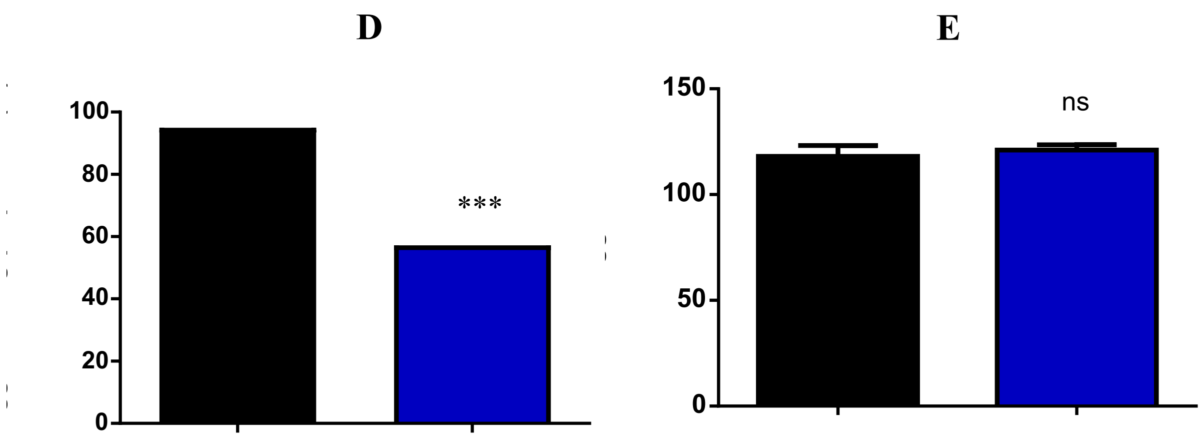

Figure S3. *C. elegans* WT and *fmo-4* KO fertility. **D**, Mean +/- SEM egg hatching in *C. elegans* at day 3 post-hatching; ***, $P < 0.001$ t test (5 df). **E**, Mean +/- SEM number of WT and *fmo-4* KO eggs within 1 day of reaching adulthood stage (ns, $p > 0.05$, t test, 5 df).

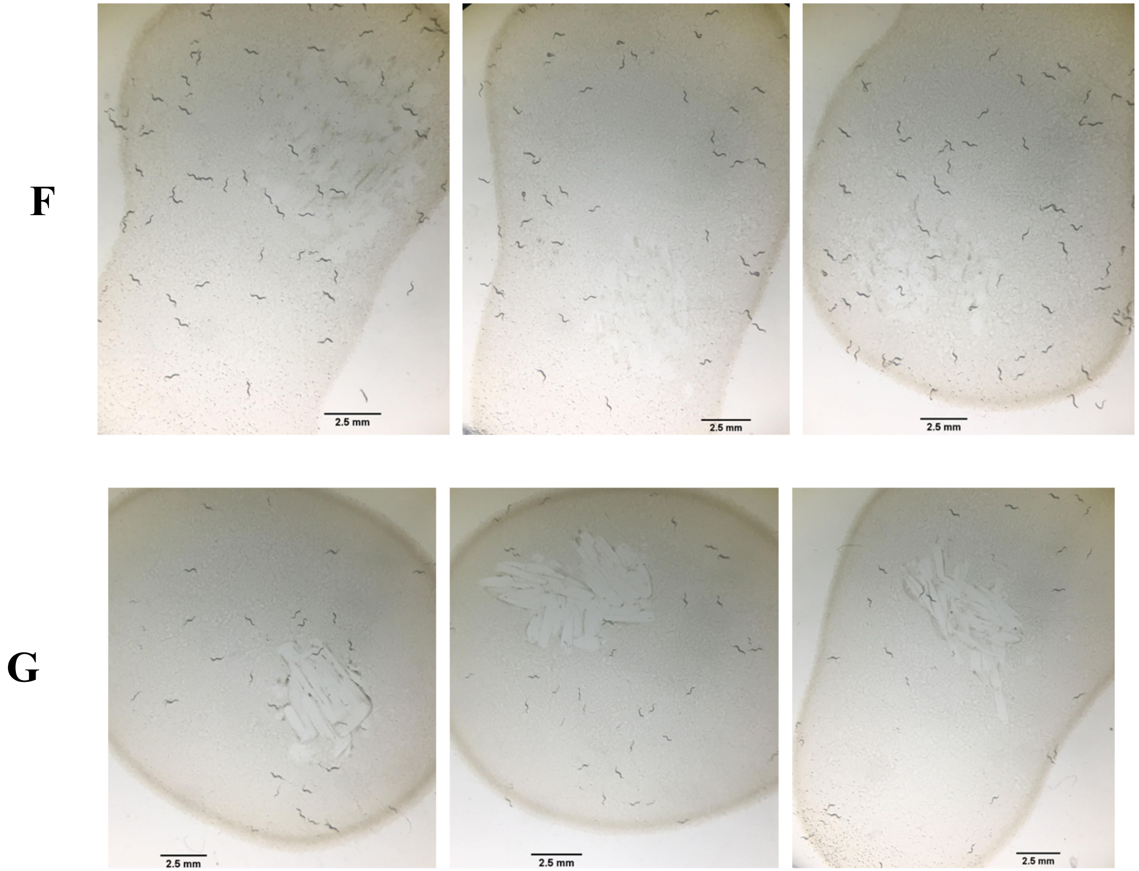

Figure S3. Images of hatched *C. elegans* worms at day 3 post-hatching (egg hatching rate of *C. elegans* wild type and *fmo-4* KO). **F**, three repeats of wild type; **G**, three repeats of *fmo-4* KO hatched worms.

A

B

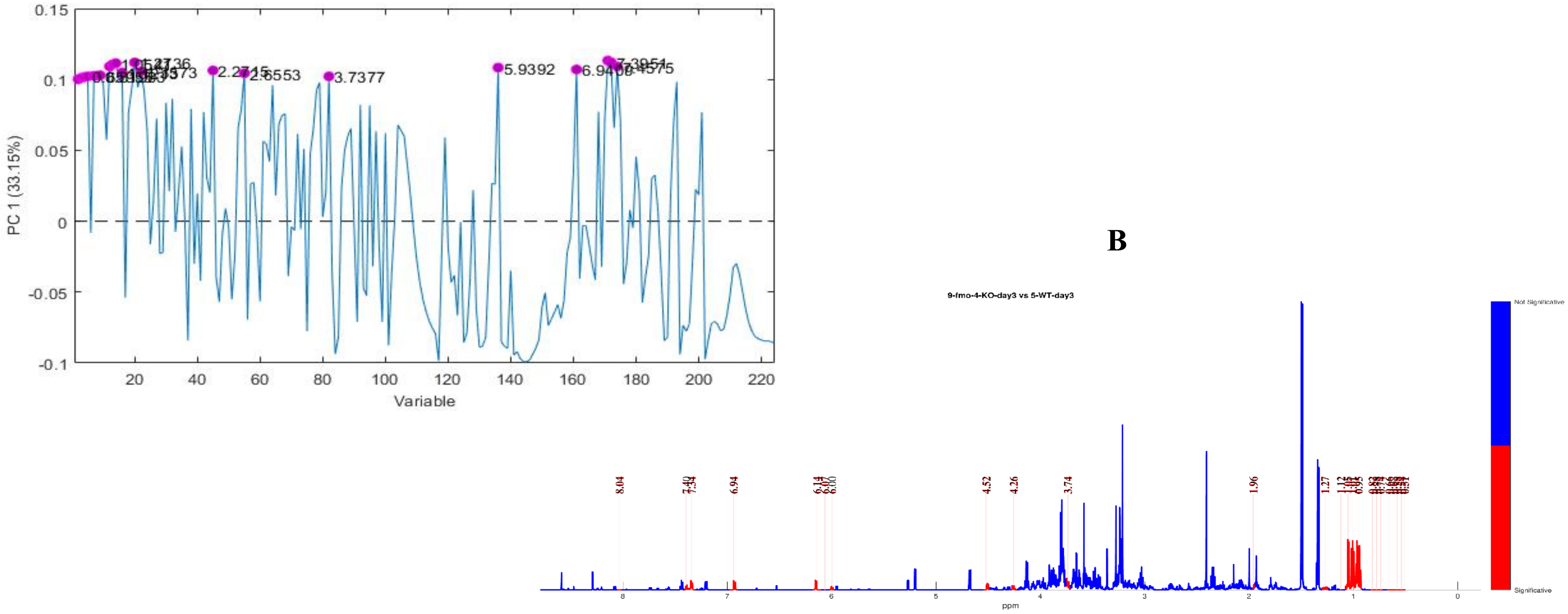

Figure S4. A, Loadings plot of day 3 post-hatching wild type and *fmo-4* KO on PC1. Purple circles corresponded to the significant buckets with their associated chemical shift in ppm. Positive score are for wild type and negative score are for *fmo-4* KO. Twenty-one buckets were significant out of a total of 224 buckets. **B,** ANOVA binary test of the 600 MHz ^{1}H NMR spectra of day 3-post-hatching wild type and *fmo-3* KO. N = 5 of each strain. The signals are colour coded by the *p*-value adjusted for an FDR of 0.05. Red peaks for those metabolite signals that are statistically significant and blue peaks for those metabolite signals that are not.

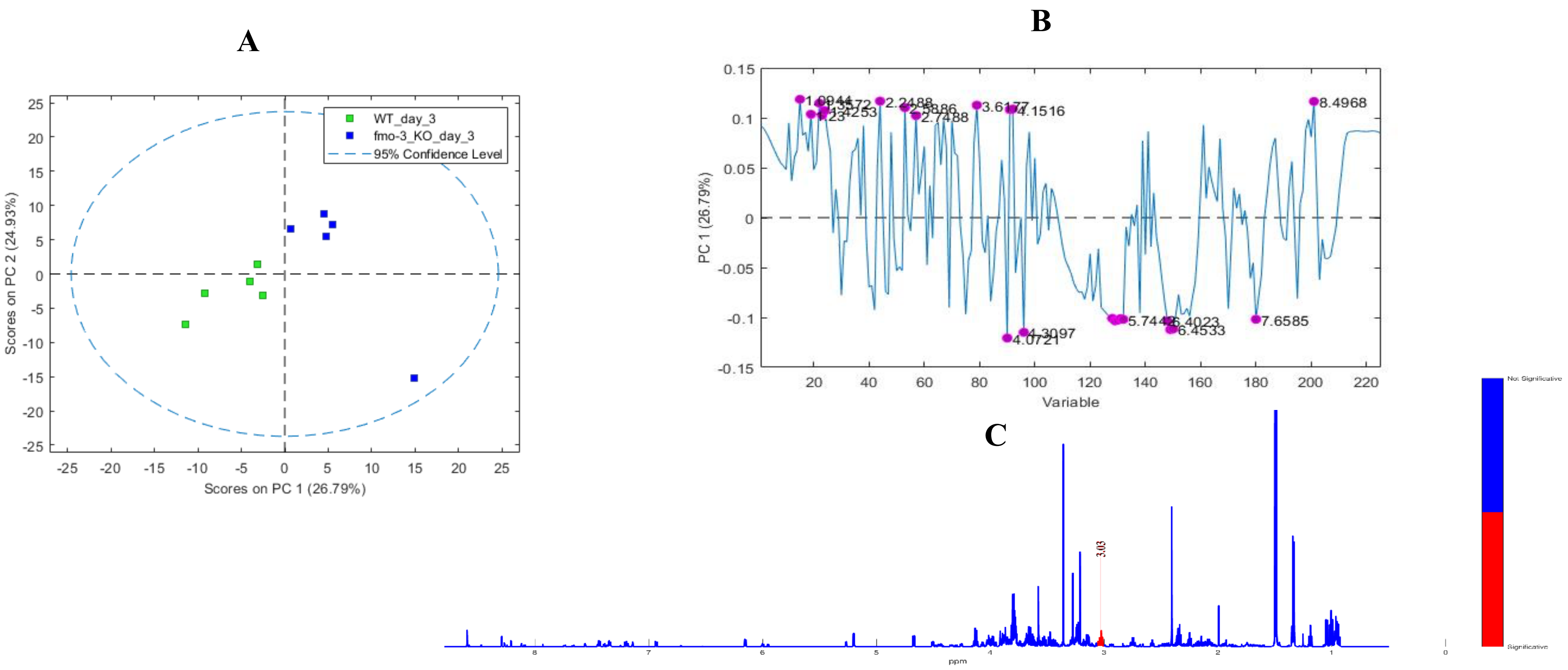

Figure S5. A, PCA of data from 600 MHz ^{1}H NMR of metabolic extract of wild type and *fmo-3* KO at day 3 post-hatching. In the two-component model, PC1 explained 27% of the total variance and PC2 explained 25%. **B,** loading scores of PCA of data from 600 MHz ^{1}H NMR of metabolic extract of wild type and *fmo-3* KO at day 3 post-hatching on PC1. Purple circles correspond to the significant buckets. Positive score are for wild type and negative score are for *fmo-3* KO. Twenty three buckets were significant of a total of 225 buckets. **C,** ANOVA binary test of the 600 MHz ^{1}H NMR spectra of day 3 post-hatching wild type and *fmo-3* KO. N = 5 of each strain. The signals are colour coded by the P-value adjusted for an FDR of 0.05.

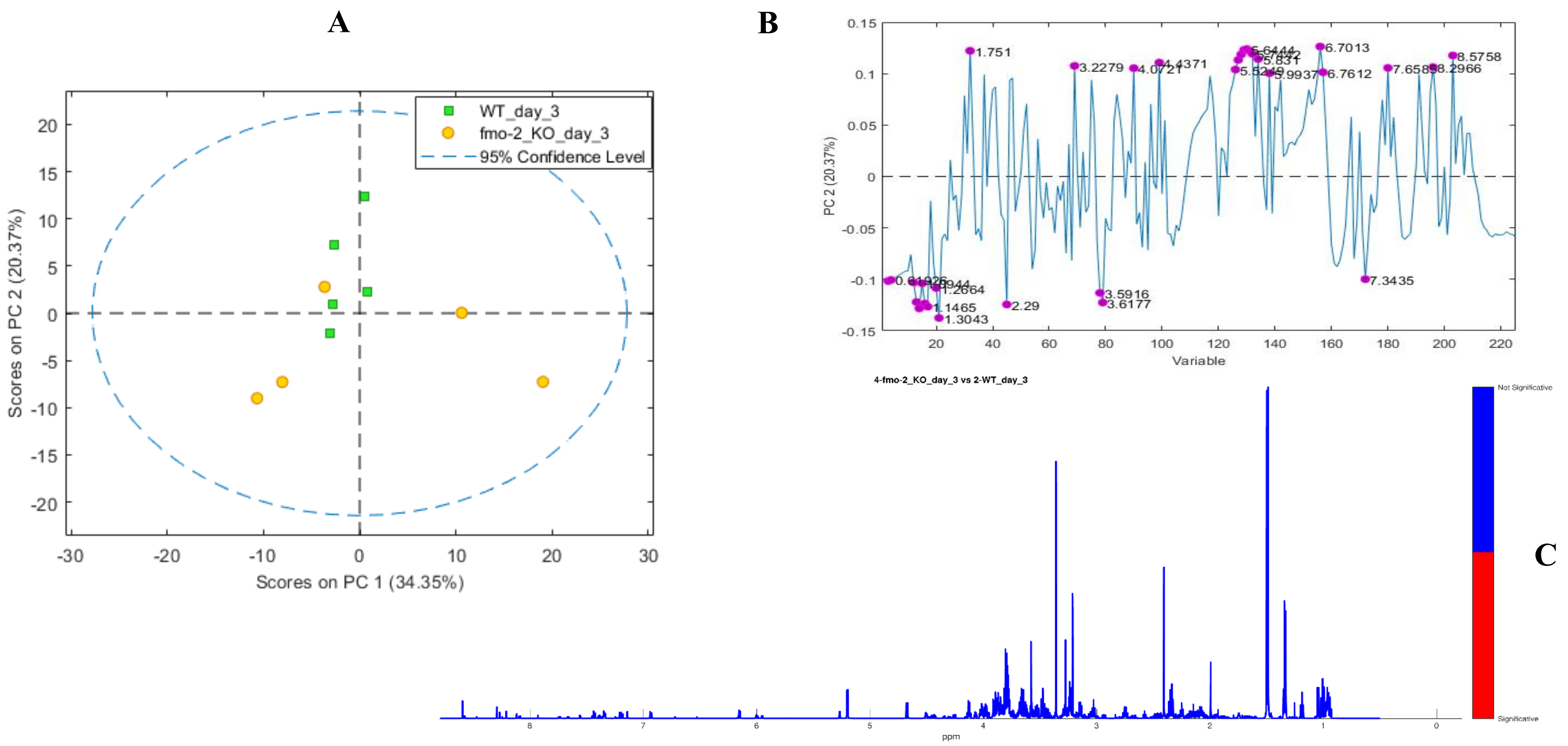

Figure S6. A, PCA of data from 600 MHz ^{1}H NMR of metabolic extract of wild type and *fmo-2* KO at day 3 post-hatching. In the two-component model, PC1 explained 34% of the total variance and PC2 explained 20%. **B,** Loading scores of wild type and *fmo-2* KO on PC2, purple circles correspond to the significant buckets. Positive score are for wild type and negative score are for *fmo-2* KO. Thirty two buckets were significant of a total of 225 buckets. **C,** ANOVA binary test of the 600 MHz ^{1}H NMR spectra of 3 days post hatching wild type and *fmo-2* KO. N = 5 of each strain. The signals are colour coded by the P-value adjusted for an FDR of 0.1.

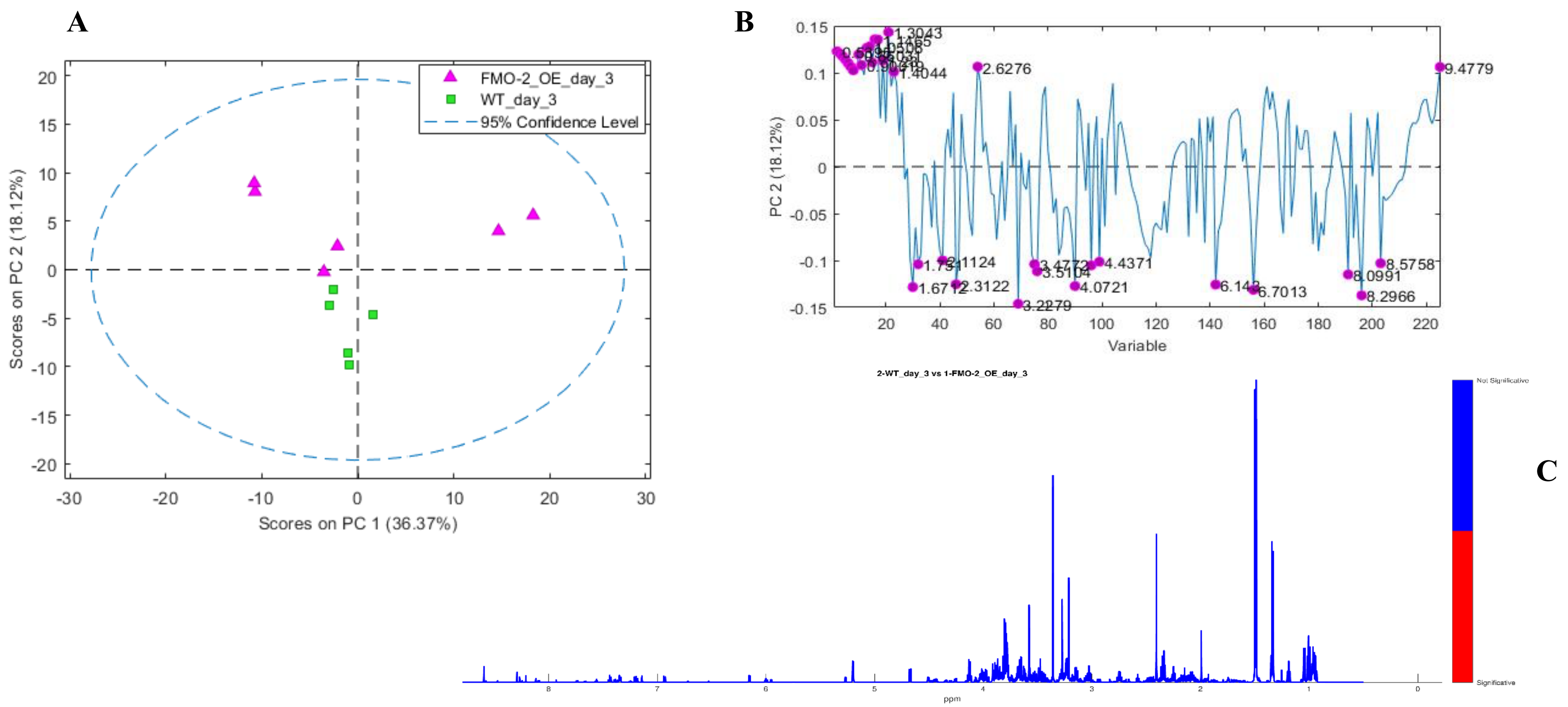

Figure S7. A, PCA of data from 600 MHz ^{1}H NMR of metabolic extract of wild type and *fmo-2* OE at day 3 post-hatching. In the two-component model, PC1 explained 36% of the total variance and PC2 explained 18%. **B,** Loading scores of wild type and *fmo-2* OE on PC2, purple circles correspond to the significant buckets. Positive score are for Wild type and negative score are for *fmo-2* OE. Thirty four buckets were significant of a total of 225 buckets. **C,** ANOVA binary test of the 600 MHz ^{1}H NMR spectra of day 3 post-hatching wild type and *fmo-2* OE. N = 5 for wild type and N=7 for *fmo-2* OE. The signals are colour coded by the P-value adjusted for an FDR of 0.1.

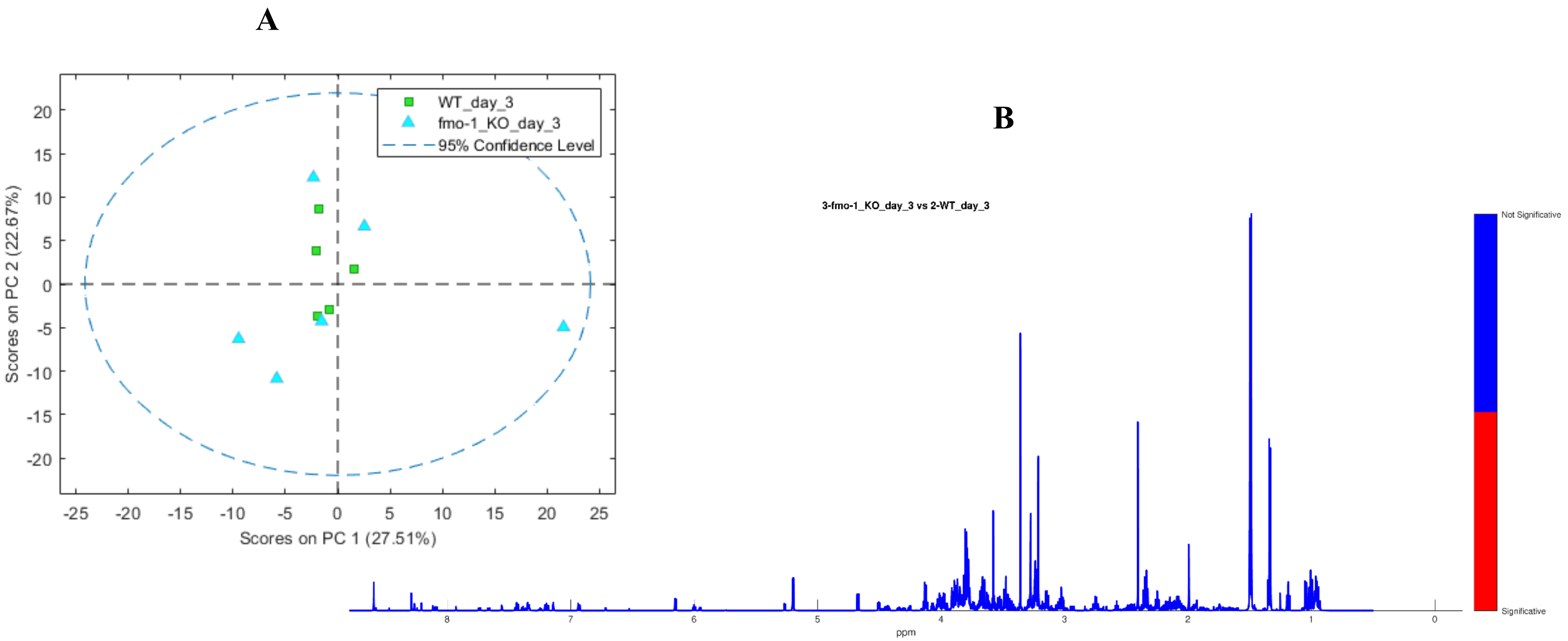

Figure S8. A, PCA of data from 600 MHz ^{1}H NMR of metabolic extract of wild type and *fmo-1* KO at day 3 post-hatching. In the two component model, PC1 explained 28% of the total variance and PC2 explained 23%. **B,** ANOVA binary test of the 600 MHz ^{1}H NMR spectra of day 3 post-hatching wild type and *fmo-1* KO. N = 5 of each strain. The signals are colour coded by the P-value adjusted for an FDR of 0.1.

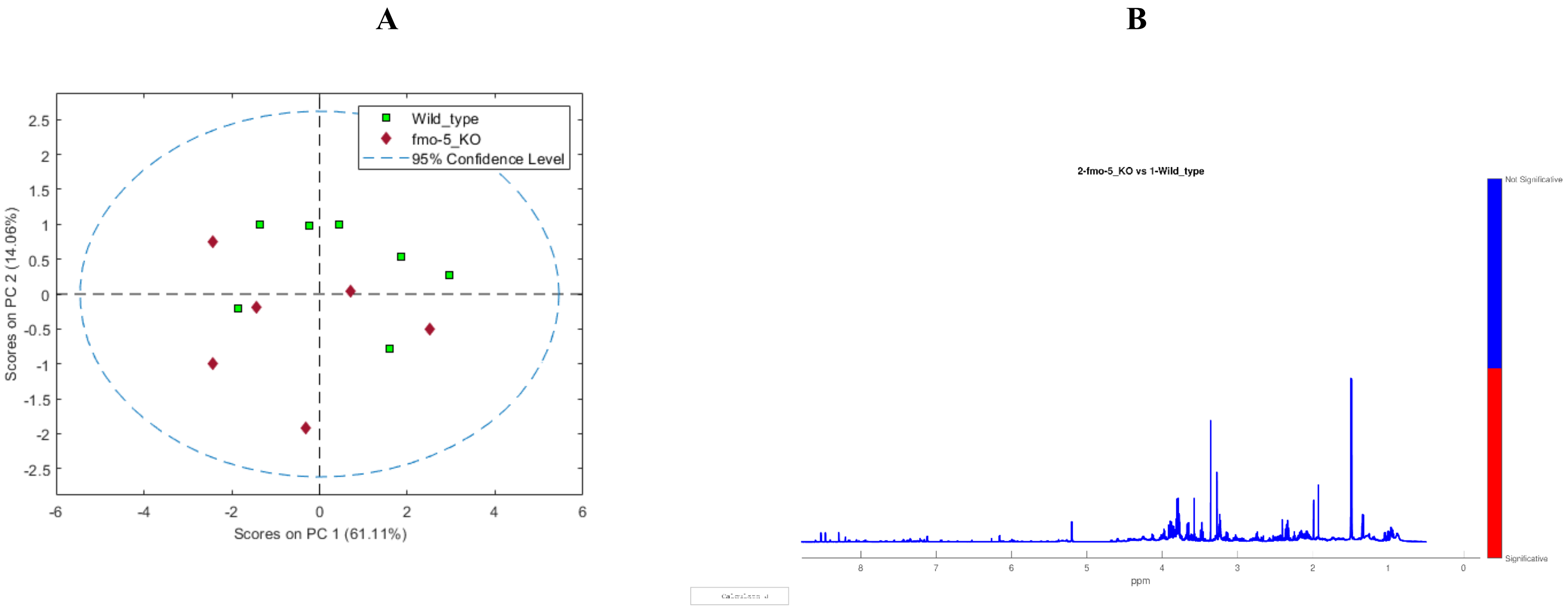

Figure S9. A, PCA of data from 600 MHz ^{1}H NMR of metabolic extract of wild type and *fmo-5* KO at day 3 post-hatching. In the two-component model, PC1 explained 61% of the total variance and PC2 explained 14%. **B,** ANOVA binary test of the 600 MHz ^{1}H NMR spectra of day 3 post-hatching wild type and *fmo-5* KO. N = 6 of each strain. The signals are colour coded by the P-value adjusted for an FDR of 0.1.

A

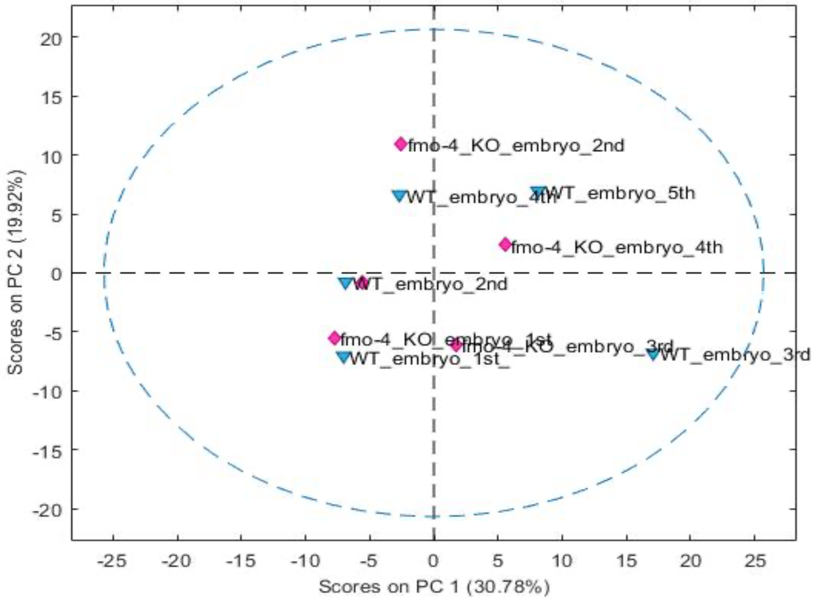

B

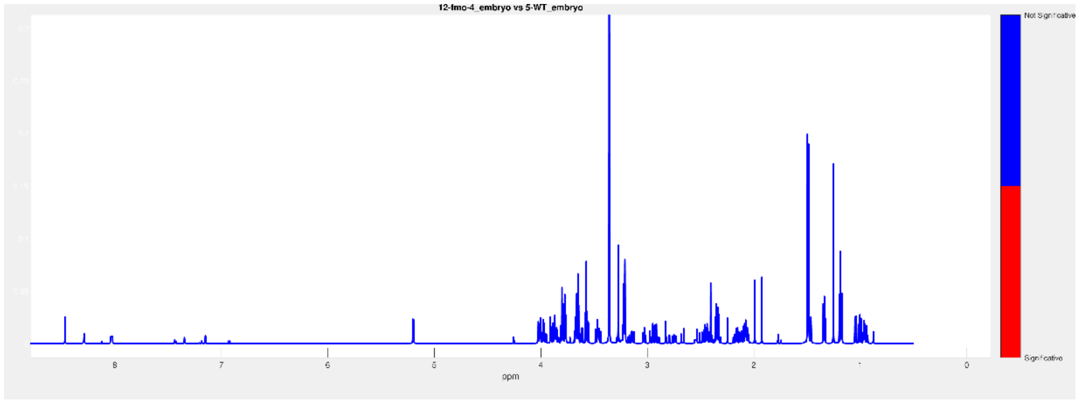

Figure S10. A, PCA of data from 600 MHz 1H NMR of metabolic extract of wild type and *fmo-4* KO *C. elegans* embryos. In the two-component model, PC1 explained 31% of the total variance and PC2 explained 20%. **B,** ANOVA binary test of the 600 MHz ^{1}H NMR spectra of wild type embryo and *fmo-4* KO embryo. N = 5 of each strain.

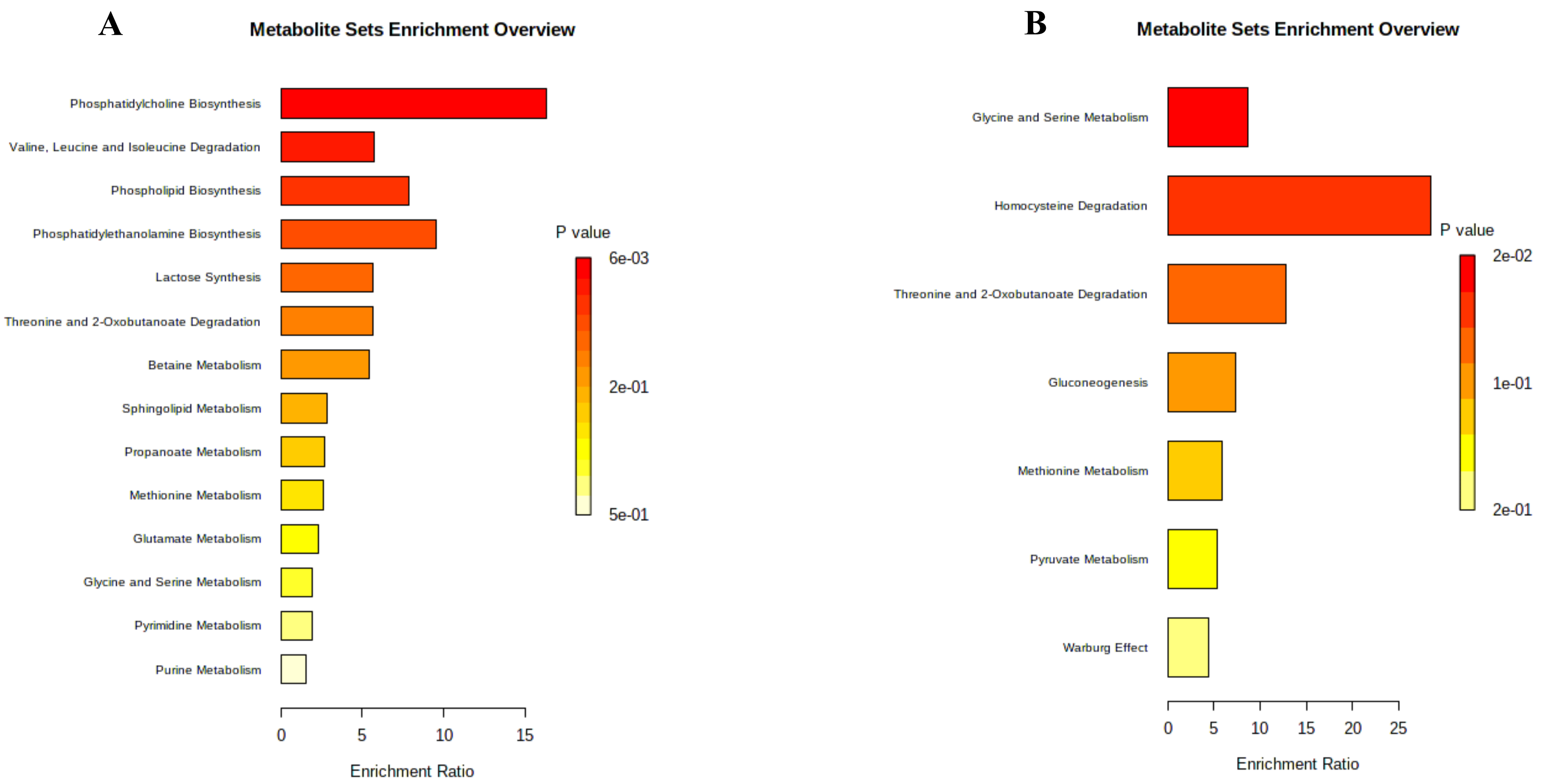

Figure S11. Metabolic pathway analyses related to the discriminating metabolites between mutants and wild type. **A**, Wild type and *fmo-2* KO at day 3 post-hatching stage; **B,** Wild type and *fmo-3* KO at day 3 post-hatching stage. The horizontal bars summarise the main metabolite sets identified in this analysis; the bars are coloured based on their p-values and the length is based on the fold enrichment.

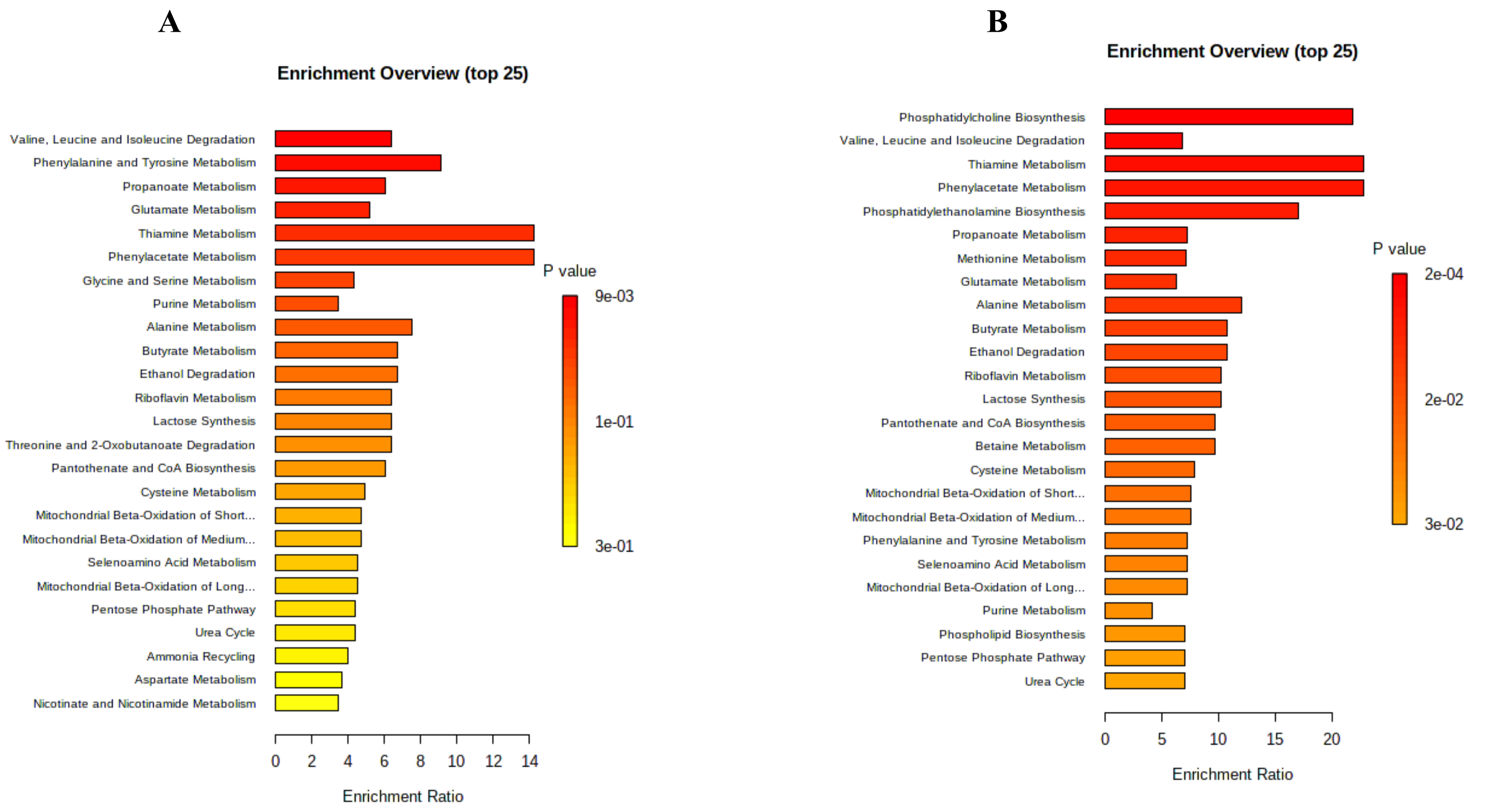

Figure S12. Metabolic pathway analyses related to the discriminating metabolites between mutants and wild type. **A**, Wild type and *fmo-4* KO day 3 post-hatching stage; **B,** Wild type and *fmo-2* OE at day 3 post-hatching stage. The horizontal bars summarise the main metabolite sets identified in this analysis; the bars are coloured based on their p-values and the length is based on the fold enrichment.

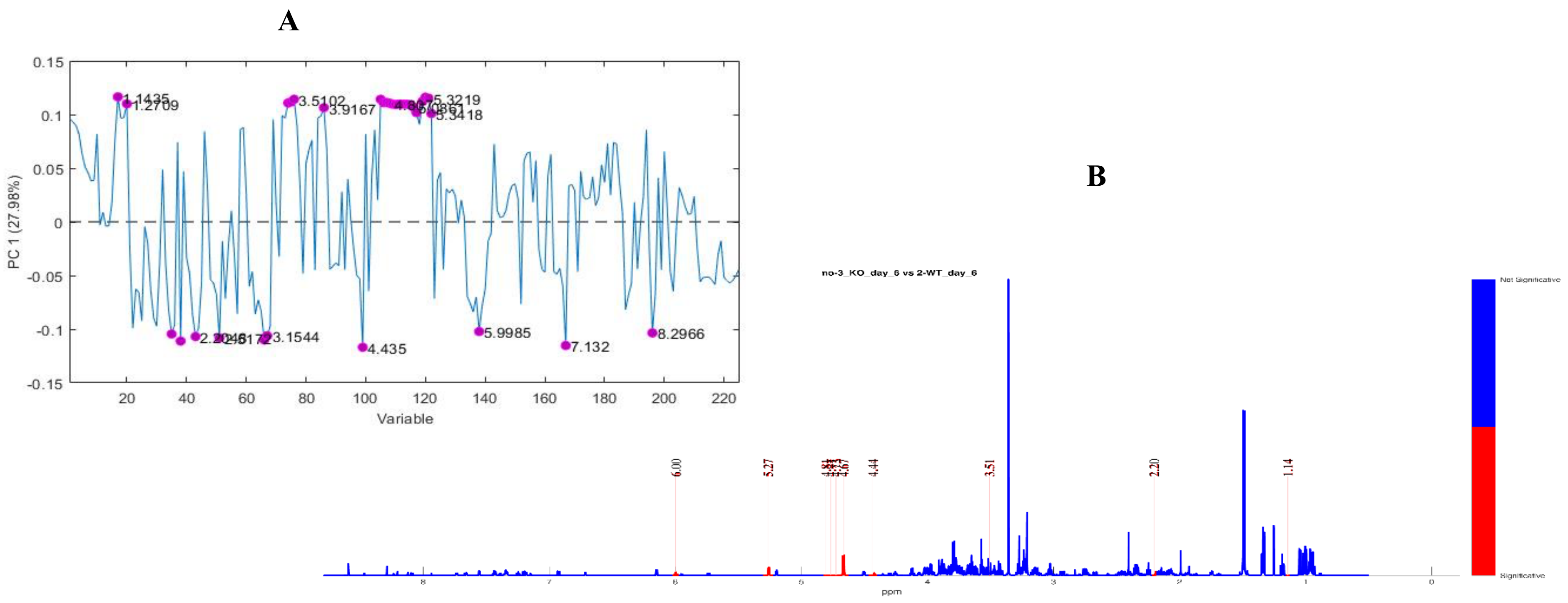

Figure S13. A, Loading scores of PCA of data from 600 MHz ^{1}H NMR of metabolic extract of wild type and *fmo-3* KO at day 6 post-hatching on PC1, purple circles correspond to the significant buckets. Positive score are for *fmo-3* KO and negative score are for wild type. Thirty-three buckets were significant of a total of 225 buckets. **B,** ANOVA binary test of the 600 MHz ^{1}H NMR spectra of day 6 post-hatching wild type and *fmo-3* KO. N = 5 of each strain. The signals are colour coded by the P-value adjusted for an FDR of 0.05. Red peaks for those metabolite signals that are more intense and significant and blue peaks for those metabolite signals that were less intense.

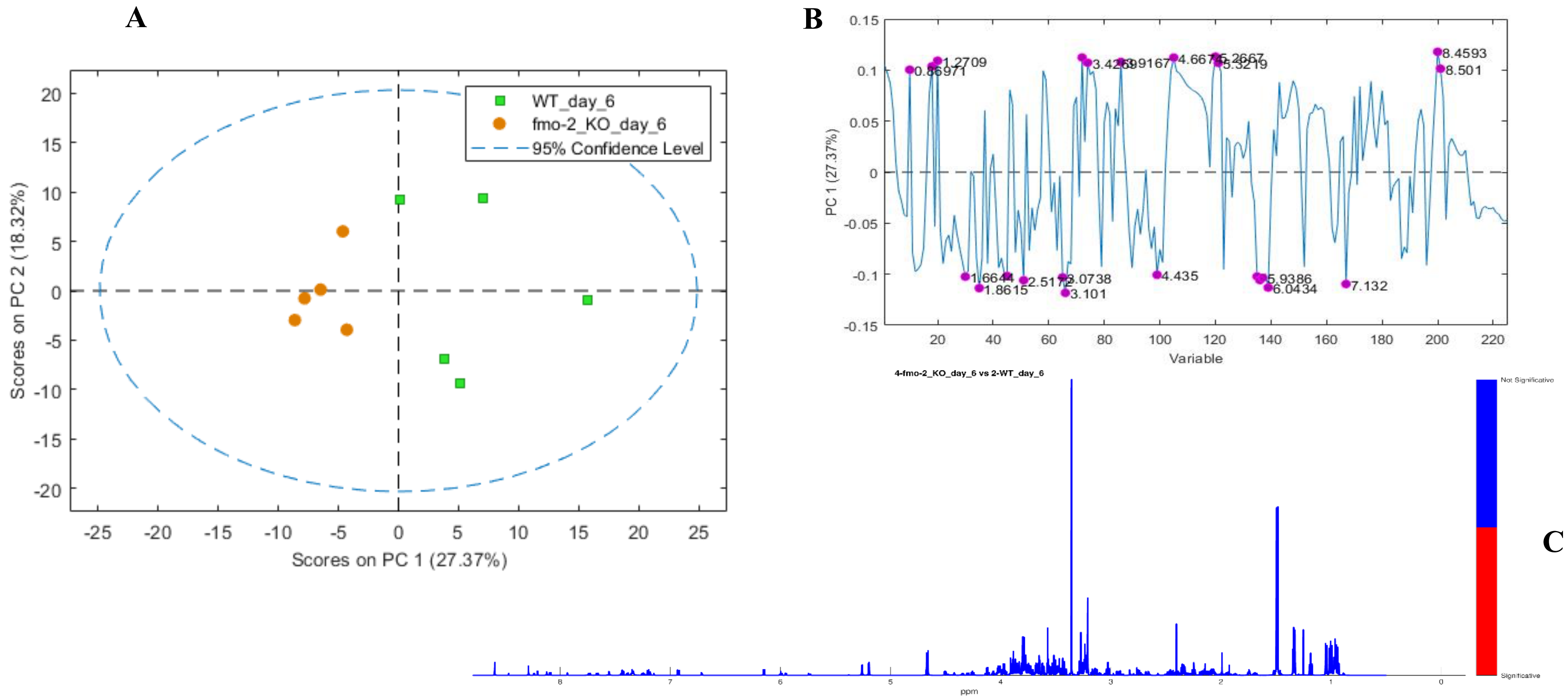

Figure S14. A, PCA of data from 600 MHz ^{1}H NMR of metabolic extract of wild type and *fmo-2* KO at day 6 post-hatching. In the two component model, PC1 explained 27% of the total variance and PC2 explained 18%. **B,** Loading scores of wild type and *fmo-2* KO on PC1, purple circles correspond to the significant buckets. Positive score are for *fmo-2* KO and negative score are for wild type. Twenty-three buckets were significant of a total of 225 buckets. **C,** ANOVA binary test of the 600 MHz ^{1}H NMR spectra of day 6 post-hatching wild type and *fmo-2* KO. N = 5 for each strain. The signals are colour coded by the P-value adjusted for an FDR of 0.1. Red peaks for those metabolite signals that are more.

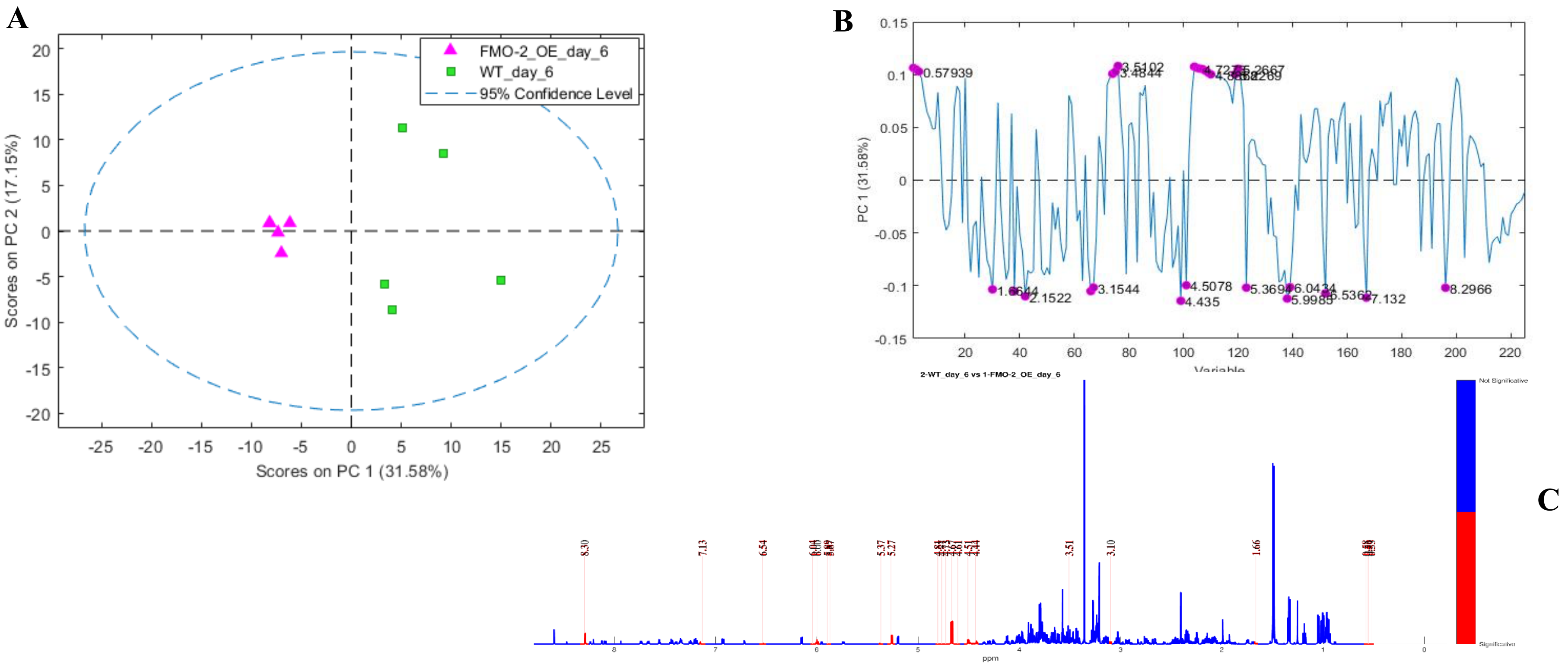

Figure S15. A, PCA of data from 600 MHz ^{1}H NMR of metabolic extract of wild type and *fmo-2* OE at day 6 post-hatching. In the two component model, PC1 explained 32% of the total variance and PC2 explained 17%. **B,** Loading scores of wild type and *fmo-2* OE on PC1, purple circles correspond to the significant buckets. Positive score are for wild type and negative score are for *fmo-2* OE. Twenty eight buckets were significant of a total of 225 buckets. **C,** ANOVA binary test of the 600 MHz ^{1}H NMR spectra of day 6 post-hatching wild type and *fmo-2* OE. N = 5 for each strain. The signals are colour coded by the P-value adjusted for an FDR of 0.1. Red peaks for those metabolite signals that are more intense and significant and blue peaks for those metabolite signals that were less intense. The fifth sample for FMO-2 OE overlaps with other FMO-2 OE samples.

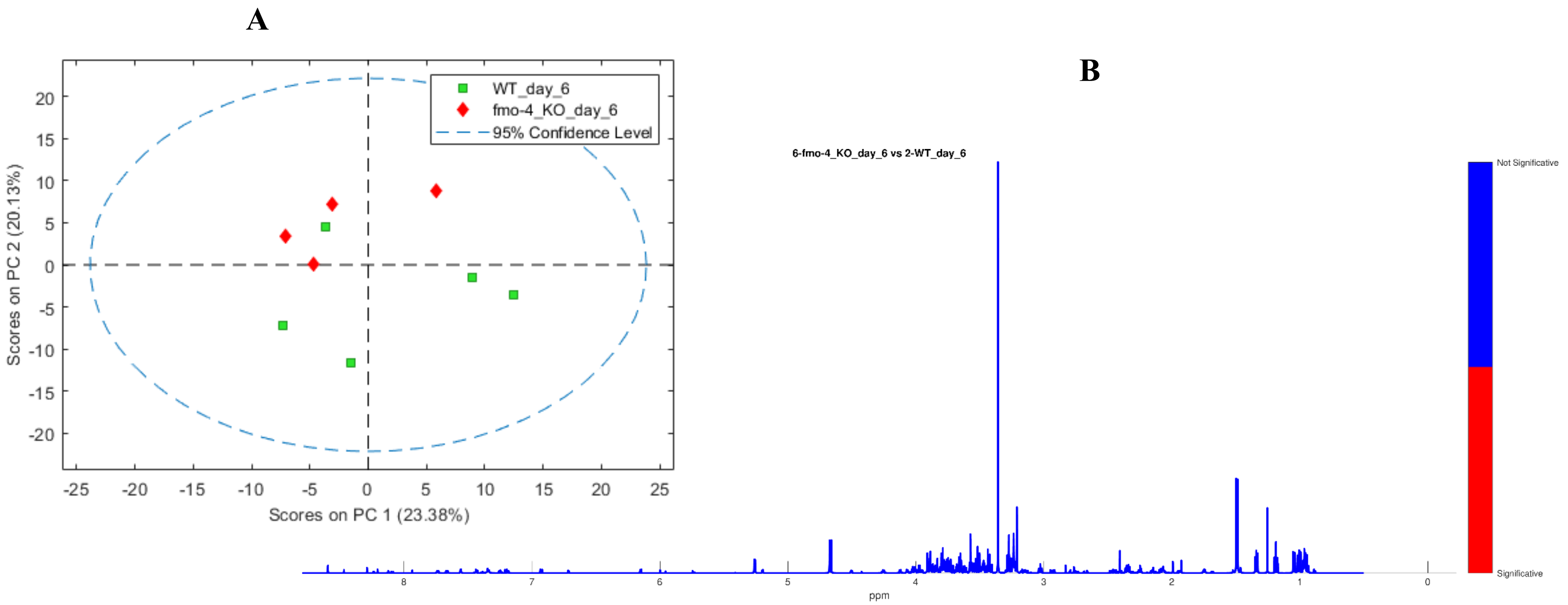

Figure S16. A, PCA of data from 600 MHz ^{1}H NMR of metabolic extract of wild type and *fmo-4* KO at day 6 post-hatching. In the two component model, PC1 explained 23% of the total variance and PC2 explained 20%. **B,** ANOVA binary test of the 600 MHz ^{1}H NMR spectra of day 6 post-hatching wild type and *fmo-4* KO. N = 5 for wild type and N=4 for *fmo-4* KO. The signals are colour coded by the P-value adjusted for an FDR of 0.1. The fifth sample for *fmo-4* KO overlaps with other *fmo-4* KO samples.

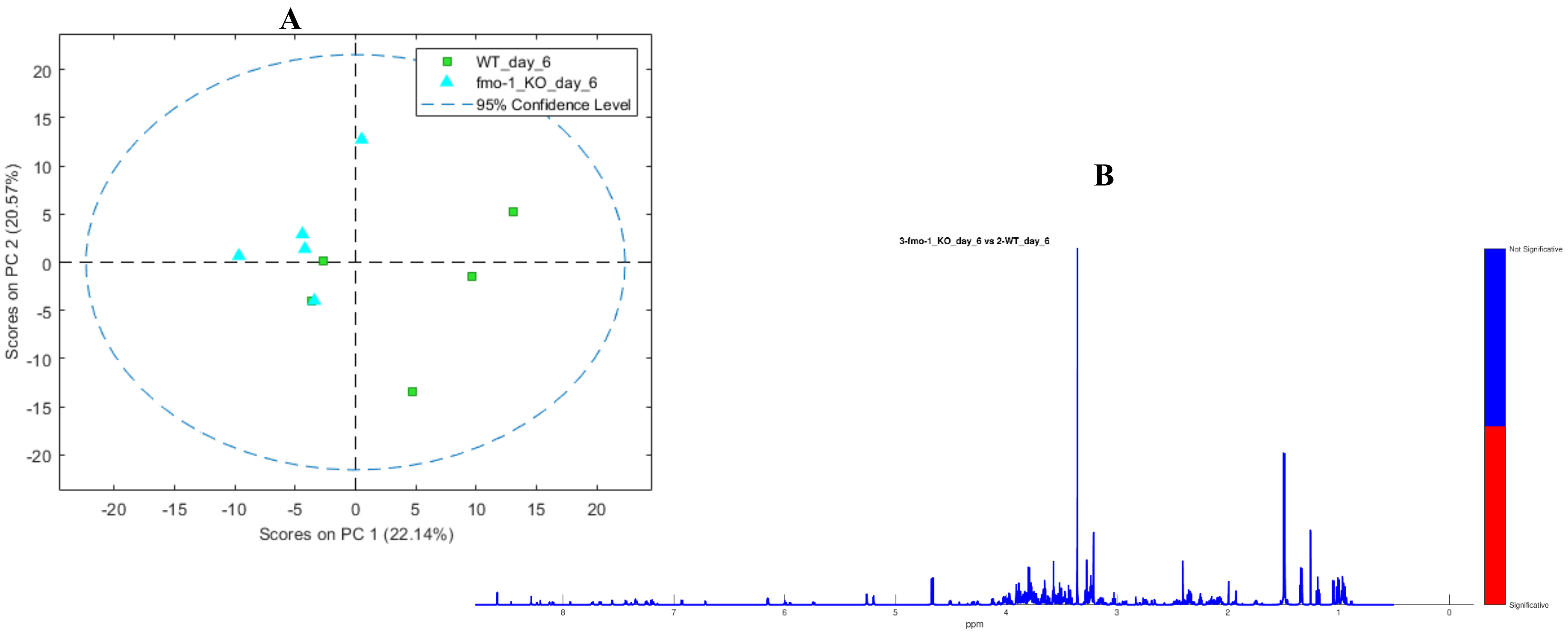

Figure S17. A, PCA of data from 600 MHz ^{1}H NMR of metabolic extract of wild type and *fmo-1* KO at day 6 post-hatching. In the two component model, PC1 explained 22% of the total variance and PC2 explained 21%. **B,** ANOVA binary test of the 600 MHz ^{1}H NMR spectra of day 6 post-hatching wild type and *fmo-1* KO. N = 5 for each strain. The signals are colour coded by the P-value adjusted for an FDR of 0.1. Red peaks for those metabolite signals that are more intense and significant and blue peaks for those metabolite signals that were less intense.

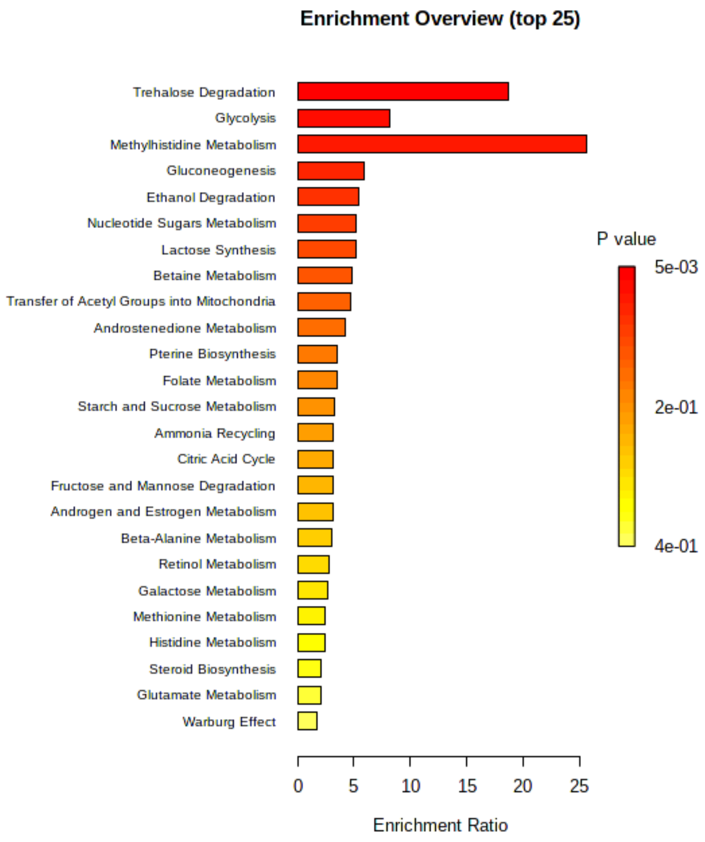

Figure S18. Metabolic pathway analyses related to the discriminating metabolites between *fmo-2* KO and wild type at day 6 post-hatching stage. The horizontal bars summarised the main metabolite sets identified in this analysis; the bars are coloured based on their P-values and the length is based on the fold enrichment.

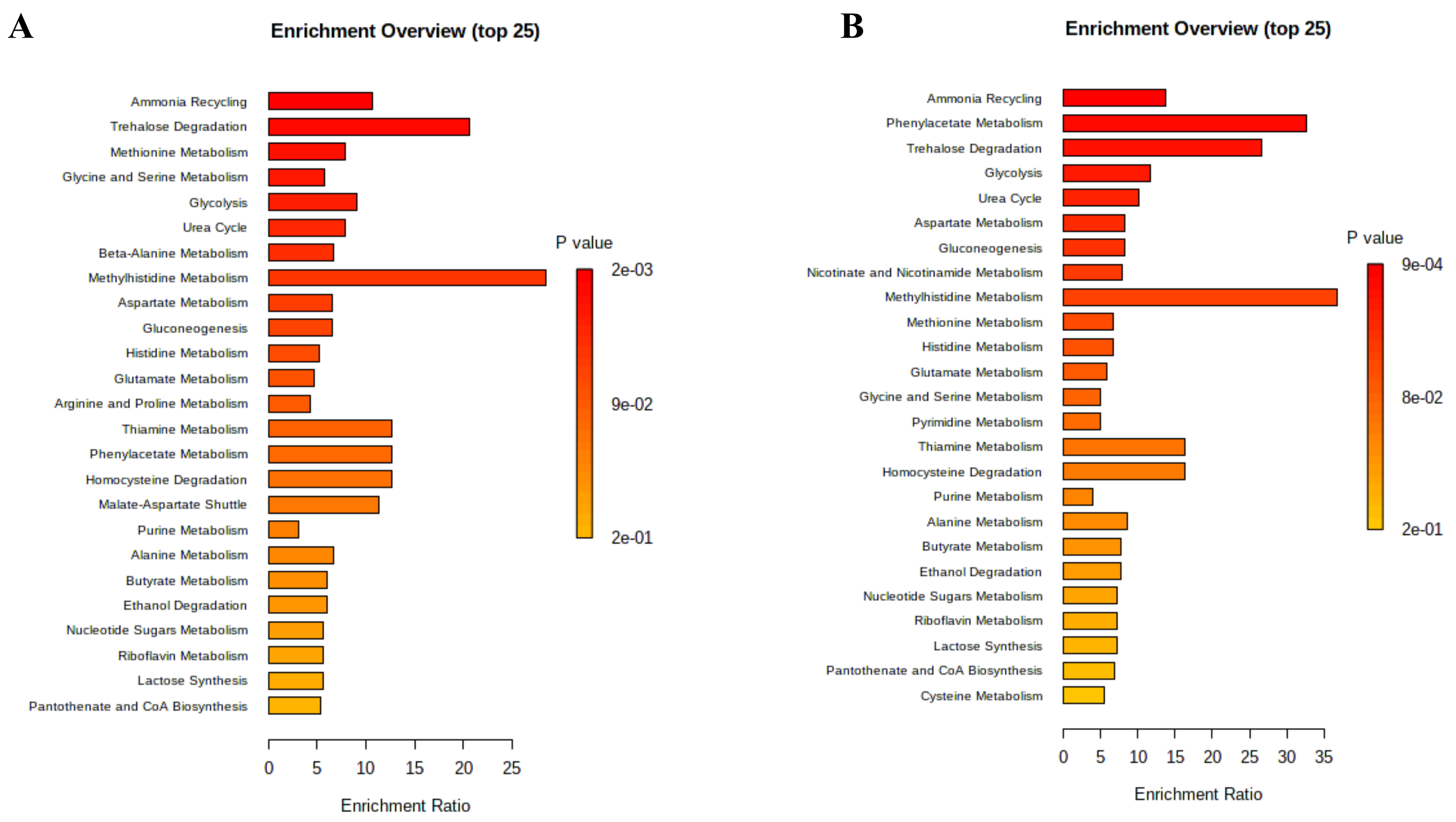

Figure S19. Metabolic pathway analyses related to the discriminating metabolites between *fmo* mutants and wild type. **A**, Wild type and *fmo-3* KO at day 6 post-hatching stage; **B,** Wild type and *fmo-2* OE at day 6 post-hatching. The horizontal bars summarised the main metabolite sets identified in this analysis; the bars are coloured based on their P-values and the length is based on the fold enrichment.

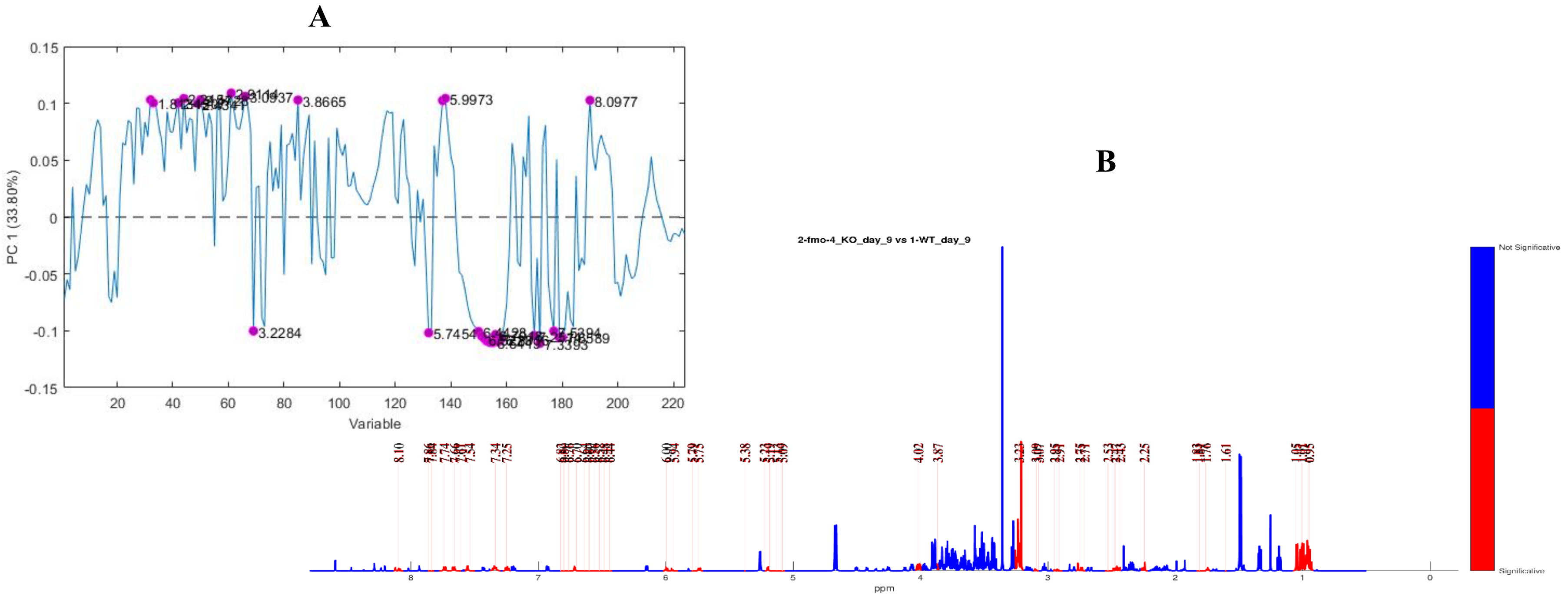

Figure S20. A, Loading scores of PCA of data from 600 MHz ^{1}H NMR of metabolic extract of wild type and *fmo-4* KO at day 9 post-hatching on PC1, purple circles correspond to the significant buckets. Positive score are for *fmo-4* KO and negative score are for wild type. Twenty eight buckets were significant of a total of 225 buckets. **B,** ANOVA binary test of the 600 MHz ^{1}H NMR spectra of day 9 post-hatching wild type and *fmo-4* KO. N = 5 of each strain. The signals are colour coded by the P-value adjusted for an FDR of 0.05. Red peaks for those metabolite signals that are more intense and significant and blue peaks for those metabolite signals that were less intense.

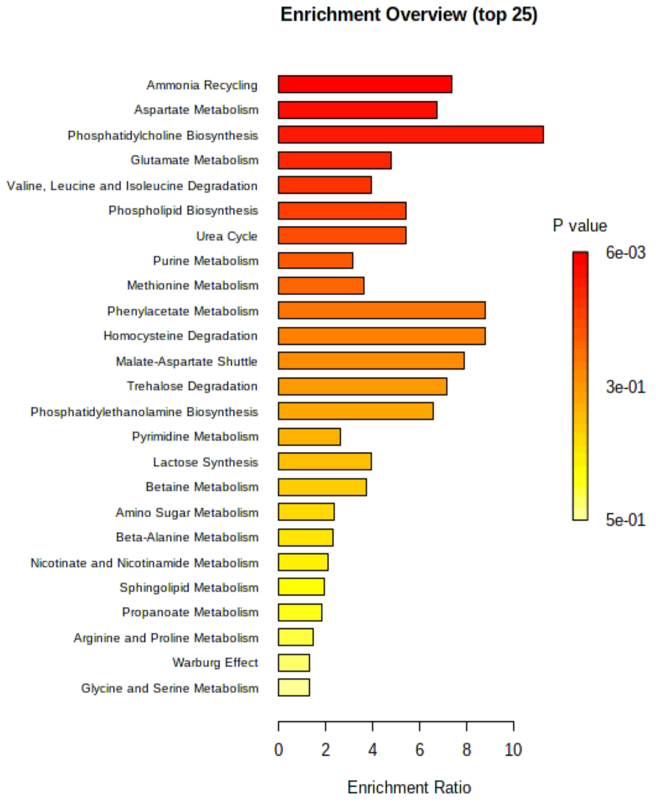

Figure S21. Metabolic pathway analyses related to the discriminating metabolites between wild type and *fmo-4* KO at day 9 post-hatching. The horizontal bars summarised the main metabolite sets identified in this analysis; the bars are coloured based on their P-values and the length is based on the fold enrichment.

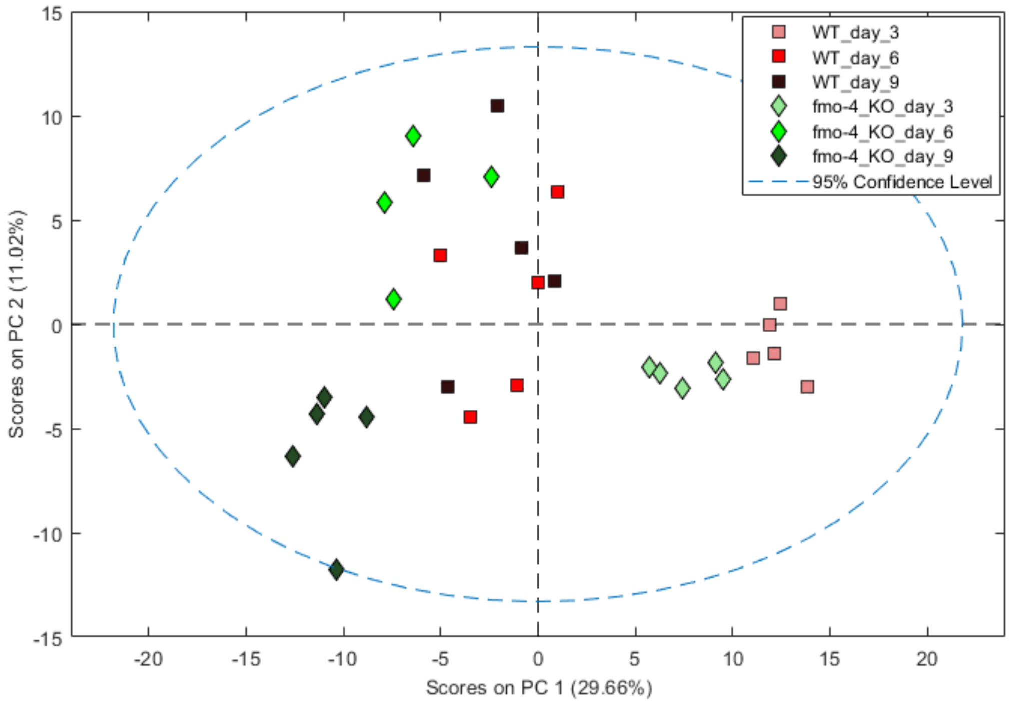

Figure S22. PCA of data from 600 MHz ^{1}H NMR of metabolic extracts of WT and *fmo-4* KO *C. elegans* adults at day 3, 6, and 9 post-hatching stages. N = 5 of each strain; one sample of the *fmo-4* KO at day 6 was an outlier.

Table S1. Summary of metabolic response of the *fmo* mutants versus WT at day 3 post-hatching stage.

**Metabolite**	**Chemical shifts (ppm)**	***fmo-3* KO vs WT Day 3**	***fmo*-4 KO vs WT Day 3**	***fmo-2* KO vs WT Day 3**	***fmo-2* OE vs WT Day 3**
Isoleucine (HMDB0000172)	0.943 (t), 1.014 (d), 3.687 (d)		↑	↑	↑
Leucine (HMDB0000687)	0.961 (d), 0.971(d)		↑	↑	↑
Valine (HMDB0000883)	1.001 (d), 1.047 (d)		↑	↑	↑
Cystathionine (HMDB0000099)	2.195 (m), 2.750 (m), 3.870 (m), 3.980 (m)	↑			
Agmatine (HMDB0001432)	1.700 (m), 3.029 (t)	↑		↓	↓
phosphoryl-choline (HMDB0001565)	4.172 (m), 3.603 (m), 3.239 (t)			↓	↓

Choline (HMDB0000097)	3.532 (m), 3.213 (t), 4.070 (m)			↓	↓
Lactate (HMDB0000190)	4.128 (q), 1.34 (d)	↑			
Threonine (HMDB0000167)	1.348 (d)	↑	↑	↑	
Phenylalanine (HMDB0000159)	7.345 (pseudo-doublet (2nd order)), 7.386 (pseudo-triplet), 7.439 (pseudo-triplet)		↑		
Tyrosine (HMDB0000158)	6.934 (pseudo-doublet), 7.203 (pseudo-doublet)		↑		
5'-UMP (HMDB0000288)	8.090 (d), 6.005 (d), 5.997 (d), 4.425 (dd)		↓	↓	↓

5'-AMP (HMDB0000045)	4.377, 4.503 (dd), 4.797 (dd), 6.151 (d), 8.290 (s), 8.590 (s)		↓	↓	↓
5'-GMP (HMDB0001397)	5.951 (d), 4.750 (m), 4.480 (m), 4.334 (m)		↓		↓
5'-ATP (HMDB0000538)	8.590 (s)		↓	↓	↓

increase	decrease

Footnotes: 5'-AMP, 5'-adenosine monophosphate; 5'-ATP, 5'-adenosine triphosphate; 5'-UMP, 5'-uridine monophosphate; 5'-GMP, 5'-guanosine monophosphate; s, singlet; d, doublet; dd, doublet of doublets; t, triplet; m, multiplet; q, quartet; ppm, parts per million. The discriminating metabolites were determined from PCA and ANOVA.

Table S2. Summary of metabolic response of the *fmo* mutants versus WT at day 6 post-hatching stage.

Metabolite	Chemical shifts (ppm)	*fmo-2* KO vs WT Day 6	*fmo-3* KO vs WT Day 6	*fmo-2* OE vs WT Day 6
alpha-D-glucose (HMDB0003345)	3.568 (m), 3.441 (m), 3.798 (m), 3.835 (m), 3.860 (m), 5.268 (d)	↓	↓	↓
beta-D-glucose (HMDB0000122)	3.281 (dd), 3.425 (dd), 3.486 (m), 3.528 (dd), 3.749 (m), 3.900 (m), 4.675 (d)	↓	↓	↓
Citrate (HMDB0000094)	2.519 (d), 2675 (d)	↑	↑	-
glutamine (HMDB0000641)	2.460 (m)	-		↑
ethanol (HMDB0000108)	1.188 (t)	↓		-
methanol (HMDB0001875)	3.360 (s)	↓	-	-
formate	8.470 (s)	↓		

(HMDB0000142)				
histidine (HMDB0000177)	7.186 (m), 8.280 (d)	↑	↑	↑
trimethylglycine (HMDB0000043)	3.914 (s br), 3.275 (m)	↓	↓	-
cystathionine-like metabolite (HMDB0000099)	2.195 (m), 2.750 (m), 3.870 (m), 3.980 (m)	-	↑	-
5′-UMP (HMDB0000288)	8.090 (d), 6.005 (d), 5.997 (d), 4.425 (dd)	↑	↑	↑
5′-AMP (HMDB0000045)	4.377, 4.503 (dd), 4.797 (dd), 6.151 (d), 8.290 (s), 8.590 (s)	-	↑	↑
5′-GMP (HMDB0001397)	5.951 (d), 4.750 (m), 4.480 (m), 4.334 (m)	↑	-	

increase	decrease

Footnotes: 5'-AMP, 5'-adenosine monophosphate; 5'-UMP, 5'-uridine monophosphate; 5'-GMP, 5'-guanosine monophosphate; br, broad signal; s, singlet; d, doublet; dd, doublet of doublets; t, triplet; m, multiplet; q, quartet; ppm, parts per million. The discriminating metabolites were determined using PCA loadings and ANOVA.

Table S3. Summary of metabolic response of the *fmo-4* KO mutant versus WT worms at day 9 post-hatching stage.

**Metabolite**	**Chemical shifts (ppm)**	***fmo*-4 KO vs WT Day 9**
Tryptophan (HMDB0000929)	7.565 (d), 7.738 (d), 7.664 (d)	↑
Trehalose (HMDB0000975)	3.830 (m), 3.894 (dd), 5.200 (d)	↓
Isoleucine (HMDB0000172)	0.943(t), 1.014(d), 3.687 (d)	↓
Leucine (HMDB0000687)	0.961 (d), 0.971 (d)	↓
Valine (HMDB0000883)	1.001 (d), 1.047 (d)	↓
Glutamine (HMDB0000641)	2.460 (m)	↓
Cystathionine (HMDB0000099)	2.195 (m), 2.750 (m), 3.870 (m), 3.980 (m)	↓
Asparagine (HMDB0000168)	2.916 (dd), 2.963 (dd)	↓
Agmatine	1.700 (m), 3.029(t)	↓

(HMDB0001432)		
Aspartate (HMDB0000191)	2.733 (dd), 2.812 (dd), 3.912 (dd)	↓
Phosphorylcholine (HMDB0001565)	4.172 (m), 3.603 (m), 3.239 (t)	↑
Choline (HMDB0000097)	3.532 (m), 3.213 (t), 4.070 (m)	↑
5′-UMP (HMDB0000288)	8.090 (d), 6.005 (d), 5.997 (d), 4.425 (dd)	↓
5′-GMP (HMDB0001397)	5.951 (d), 4.750 (m), 4.480 (m), 4.334 (m)	↓
increase	decrease	

Footnotes: 5'-UMP, 5'-uridine monophosphate; 5'-GMP, 5'-guanosine monophosphate; br, broad signal; s, singlet; d, doublet; dd, doublet of doublets; t, triplet; m, multiplet; q, quartet; ppm, parts per million. The discriminating metabolites were determined using PCA and ANOVA.

Table S4. Comparison of the significant bucket of wild type at day 3 post-hatching versus wild type at day 6 post-hatching (ANOVA).

ANOVA bucket Number	Metabolite	ppm	P-value	FDR-controlled P-value	Mean WT day3	Mean WT day6
20	unknown	1.271	0.0007	0.0073	0.38	1.50
25	alanine	1.477	0.0050	0.0283	12.02	7.11
38	unknown singlet	1.991	0.0009	0.0079	1.08	0.56
39	unknown	2.028	0.0038	0.0228	0.30	0.17
43	cystathionine like metabolite	2.205	0.0001	0.0020	0.38	0.11
47	succinate	2.407	0.0110	0.0455	2.40	1.06
52	beta alanine	2.588	0.0000	0.0001	0.45	0.04
62	asparagine	2.983	0.0033	0.0210	0.25	0.08
63	agmatine	3.038	0.0007	0.0073	1.58	1.06
73	beta-D-glucose	3.428	0.0025	0.0166	1.07	2.65
80	alpha-D-glucose	3.711	0.0105	0.0440	0.12	0.22
81	alpha-D-glucose	3.735	0.0013	0.0104	0.61	1.59
82	alanine	3.786	0.0016	0.0126	7.60	4.65
91	lactate	4.133	0.0069	0.0353	1.04	0.47
98	5'-UMP	4.431	0.0000	0.0001	0.51	0.08
100	5'-AMP	4.508	0.0002	0.0039	0.65	0.34
104	beta-D-glucose	4.667	0.0000	0.0015	0.52	1.88
119	alpha-D-glucose	5.268	0.0003	0.0051	0.32	0.88
136	5'-UMP	5.998	0.0004	0.0051	0.28	0.13
165	histidine	7.131	0.0000	0.0012	0.24	0.06
194	5’-AMP	8.295	0.0001	0.0019	0.50	0.21
201	5'-AMP/5'-ATP	8.576	0.0122	0.0493	0.47	0.29

Table S5. Comparison of the significant bucket of wild type at day 3 post-hatching versus wild type at day 9 post-hatching (ANOVA).

ANOVA bucket Number	Metabolites	ppm	P-vlaue	FDR-controlled P-value	Mean WT day3	Mean WT day9
20	unknown	1.271	0.0016	0.0109	0.38	1.03
22	lactate/threonine	1.358	0.0119	0.0409	4.25	2.17
25	alanine	1.477	0.0002	0.0032	12.02	5.62
30	agmatine	1.669	0.0012	0.0096	0.60	0.17
38	unknown	1.991	0.0000	0.0022	1.08	0.40
41	glutamate	2.111	0.0002	0.0032	0.31	0.11
42	glutathione/unknown	2.152	0.0051	0.0241	1.68	0.87
43	cystathionine like metabolite	2.205	0.0000	0.0011	0.38	0.09
45	unknown	2.297	0.0126	0.0424	0.50	0.30
46	unknown	2.353	0.0001	0.0032	3.05	1.54
47	succinate	2.407	0.0029	0.0161	2.40	0.78
52	beta alanine	2.588	0.0000	0.0000	0.45	0.02
56	cystathionone like metabolite	2.753	0.0002	0.0032	1.17	0.59
62	asparagine	2.983	0.0052	0.0241	0.25	0.11
63	agmatine	3.038	0.0001	0.0025	1.58	0.67
64	unknown	3.077	0.0015	0.0106	0.04	0.01
66	unknown	3.138	0.0008	0.0075	1.49	0.68
67	beta alanine	3.182	0.0047	0.0228	0.77	0.33
73	beta D-glucose	3.428	0.0001	0.0025	1.07	3.49
74	beta-D-glucose	3.484	0.0037	0.0195	1.57	3.25
80	alpha-D-glucose	3.711	0.0003	0.0041	0.12	0.34
81	alpha-D-glucose	3.735	0.0012	0.0096	0.61	2.42

82	alanine	3.786	0.0016	0.0109	7.60	4.48
91	lactate	4.133	0.0012	0.0096	1.04	0.31
100	5'-AMP	4.508	0.0077	0.0319	0.65	0.39
104	beta-D-glucose	4.667	0.0005	0.0057	0.52	2.62
117	trehalose	5.185	0.0093	0.0350	1.30	0.52
119	alpha-D-glucose	5.268	0.0005	0.0053	0.32	1.25
140	5'-AMP	6.141	0.0019	0.0126	0.50	0.29
165	histidine	7.131	0.0000	0.0011	0.24	0.05
168	unknown	7.253	0.0093	0.0350	0.13	0.26
171	phenylalanine	7.394	0.0069	0.0292	0.19	0.09
172	phenylalanine	7.434	0.0033	0.0179	0.52	0.31
194	5'-AMP	8.295	0.0002	0.0032	0.50	0.26
201	5'-AMP/5'-ATP	8.576	0.0008	0.0074	0.47	0.25

Mean represents the peak area

Table S6. Comparison of the significant bucket of *fmo-4* KO at day 3 post-hatching versus *fmo-4* KO at day 6 post-hatching (ANOVA).

**ANOVA bucket number**	**Metabolites**	**ppm**	**P-value**	**FDR-controlled P-value**	**Mean *fmo-4* KO day 6**	**Mean *fmo-4* KO day 3**
bucket no	metabolites	ppm	p vlaue	FDR	mean day6	mean day3
19	unknown	1.248	0.0047	0.0486	2.58	0.31
28	unknown	1.602	0.0039	0.0436	0.02	0.11
38	isoleucine/unknown	1.990	0.0001	0.0020	0.40	1.05
41	glutamate	2.111	0.0048	0.0486	0.08	0.25
43	cystathionine	2.205	0.0002	0.0046	0.07	0.33
48	succinate	2.404	0.0015	0.0256	0.80	2.74
53	beta alanine	2.586	0.0000	0.0013	0.01	0.47
67	unknown	3.144	0.0006	0.0128	0.73	1.25
74	beta-D-glucose	3.427	0.0036	0.0428	5.19	1.01
81	alpha-D-glucose	3.711	0.0020	0.0301	0.50	0.08
99	5'-UMP	4.433	0.0000	0.0013	0.11	0.54
101	5'-AMP	4.509	0.0029	0.0383	0.28	0.72
105	beta-D-glucose	4.669	0.0013	0.0243	5.81	0.66
118	trehalose	5.187	0.0000	0.0014	0.22	1.14
167	histidine or unknown singlet	7.129	0.0000	0.0000	0.01	0.24
196	5'-AMP	8.297	0.0007	0.0151	0.21	0.54

Mean represents the peak area

Table S7. Comparison of the significant bucket of *fmo-4* KO at day 3 post-hatching versus *fmo-4* KO at day 9 post-hatching (ANOVA).

**ANOVA bucket number**	**Metabolites**	**ppm**	**P-value**	**FDR-controlled P-value**	**Mean *fmo-4* KO day9**	**Mean *fmo-4* KO day3**
18	ethanol	1.189	0.0065	0.0175	2.09	0.94
19	unknown	1.248	0.0004	0.0018	1.89	0.31
22	lactate/threonine	1.359	0.0017	0.0064	2.05	5.50
25	alanine	1.478	0.0000	0.0003	5.44	11.43
38	isoleucine/unknown	1.990	0.0000	0.0000	0.21	1.05
41	glutamate	2.111	0.0015	0.0062	0.03	0.25
42	unknown	2.152	0.0020	0.0068	0.33	1.43
43	cystathionine	2.205	0.0001	0.0006	0.03	0.33
44	valine	2.250	0.0001	0.0008	0.35	0.97
47	glutamate	2.350	0.0002	0.0009	1.27	2.78
48	succinate	2.404	0.0018	0.0065	0.84	2.74
49	glutamine	2.447	0.0112	0.0256	0.15	0.46
51	citrate	2.524	0.0250	0.0480	0.00	0.11
53	beta alanine	2.586	0.0000	0.0002	0.01	0.47
61	asparagine	2.906	0.0000	0.0002	0.01	0.26
62	asparagine	2.950	0.0001	0.0006	0.02	0.20
64	agmatine	3.040	0.0000	0.0000	0.53	1.57
67	unknown	3.144	0.0000	0.0000	0.31	1.25
68	beta alanine	3.182	0.0039	0.0114	0.23	0.69
69	choline/phosphorylcholine	3.233	0.0000	0.0001	12.02	5.90
70	trimethylglycine/beta-D-glucose	3.290	0.0238	0.0462	3.21	2.45
72	methanol	3.357	0.0017	0.0064	9.56	3.97
74	beta-D-glucose	3.427	0.0001	0.0006	3.67	1.01

75	alpha-D-glucose	3.484	0.0002	0.0012	3.02	1.27
77	choline/ alpha-D-glucose	3.552	0.0045	0.0128	5.50	3.31
81	alpha-D-glucose	3.711	0.0001	0.0008	0.31	0.08
82	leucine	3.740	0.0010	0.0041	2.88	0.93
83	arginine/ alpha-D-glucose	3.793	0.0002	0.0012	3.46	6.34
86	trimethylglycine	3.918	0.0001	0.0008	2.10	1.04
88	phenylalanine CH	3.989	0.0021	0.0072	0.07	0.20
89	phenylalanine CH	4.021	0.0015	0.0062	0.88	1.61
90	choline	4.074	0.0069	0.0177	1.13	0.41
91	lactate	4.124	0.0113	0.0256	0.19	0.98
99	5'-UMP	4.433	0.0001	0.0009	0.17	0.54
100	5'-GMP	4.468	0.0057	0.0157	0.00	0.00
101	5'-AMP	4.509	0.0005	0.0021	0.39	0.72
105	beta-D-glucose	4.669	0.0001	0.0008	3.22	0.66
107	5'-GMP	4.768	0.0175	0.0360	0.00	0.00
108	5'-AMP	4.808	0.0127	0.0278	0.00	0.00
118	trehalose	5.187	0.0000	0.0002	0.09	1.14
120	alpha-D-glucose	5.267	0.0000	0.0002	1.37	0.39
132	unknown	5.742	0.0004	0.0019	0.54	0.19
137	5'-GMP	5.939	0.0000	0.0005	0.03	0.17
138	5'-UMP	5.999	0.0026	0.0082	0.13	0.28
167	histidine or unknown singlet	7.129	0.0000	0.0000	0.01	0.24
170	tryptophan	7.255	0.0000	0.0003	0.83	0.24
172	phenylalanine/overlapped with another metabolite	7.339	0.0019	0.0068	1.24	0.86
173	phenylalanine	7.395	0.0069	0.0177	0.06	0.19
174	phenylalanine	7.435	0.0104	0.0245	0.19	0.53
178	tryptophan	7.574	0.0002	0.0010	0.72	0.25

180	analogue of tryptophan	7.658	0.0000	0.0002	0.74	0.25
182	tryptophan	7.739	0.0002	0.0011	0.73	0.30
191	5'-UMP	8.098	0.0119	0.0268	0.12	0.34
196	5'-AMP	8.297	0.0003	0.0016	0.24	0.54
200	formate	8.457	0.0124	0.0275	0.12	0.02
203	5'-AMP/5'-ATP	8.576	0.0074	0.0185	0.35	0.48

Mean represents the peak area

Table S8. Comparison of the significant bucket of *fmo-1* KO at day 3 post-hatching versus *fmo-1* KO at day 6 post-hatching (ANOVA).

**ANOVA bucket number**	**Metabolites**	**ppm**	**P-value**	**FDR-controlled P-value**	**Mean *fmo-1* KO day6**	**Mean *fmo-1* KO day3**
20	isoleucine	1.275	0.0007	0.0253	1.32	0.46
26	unknown	1.535	0.0010	0.0308	0.38	0.12
63	unknown	2.983	0.0019	0.0462	0.08	0.25
65	unknown	3.072	0.0004	0.0251	0.05	0.20
68	beta alanine	3.193	0.0006	0.0253	0.08	0.29
74	beta D-glucose /alpha-D-glucose	3.423	0.0001	0.0063	1.56	0.61
81	alpha-D-glucose	3.713	0.0016	0.0457	0.16	0.07
105	beta-D-glucose	4.667	0.0000	0.0011	1.88	0.48
120	alpha-D-glucose	5.273	0.0001	0.0082	0.64	0.24

Mean represents the peak area

Table S9. Comparison of the significant bucket of *fmo-2* KO at day 3 post-hatching versus *fmo-2* KO at day 6 post-hatching (ANOVA).

**ANOVA bucket number**	**Metabolites**	**ppm**	**P-value**	**FDR-controlled P-value**	**Mean *fmo-2* KO day6**	**Mean *fmo-2* KO day3**
48	succinate	2.407	0.0004	0.0096	1.35	3.11
64	agmatine	3.033	0.0001	0.0093	0.91	1.46
91	asparagine	4.101	0.0003	0.0096	0.05	0.02
169	unknown	7.237	0.0003	0.0096	0.16	0.05
170	unknown	7.263	0.0001	0.0093	0.05	0.01

Mean represents the peak area

Table S10. Comparison of the significant bucket of *fmo-3* KO at day 3 post-hatching versus *fmo-3* KO at day 6 post-hatching (ANOVA).

ANOVA bucket number	Metabolites	ppm	P-value	FDR-controlled P-value	Mean *fmo-3* KO day6	Mean *fmo-3* KO day3
47	glutamate	2.373	0.0003	0.0189	0.30	0.40
48	succinate	2.407	0.0002	0.0184	1.02	2.95
52	beta alanine	2.576	0.0009	0.0217	0.18	0.46
61	asparagine	2.927	0.0025	0.0380	0.45	0.31
64	agmatine	3.048	0.0000	0.0006	1.03	2.07
67	beta alanine	3.178	0.0004	0.0189	0.47	0.77
69	agmatine	3.253	0.0011	0.0239	3.53	6.08
78	threonine	3.619	0.0035	0.0443	0.91	1.44
118	trehalose	5.208	0.0019	0.0308	0.23	0.61
168	tryptophan/unknown	7.247	0.0005	0.0189	0.24	0.03
179	tryptophan	7.663	0.0041	0.0443	0.24	0.05

Mean represents the peak area

Table S11. Comparison of the significant bucket of *fmo-2* OE at day 3 post-hatching versus *fmo-2* OE at day 6 post-hatching (ANOVA).

ANOVA bucket number	Metabolites	ppm	P-value	FDR-controlled P-value	Mean *fmo-2* OE day6	Mean *fmo-2* OE day3
20	unknown singlet	1.266	0.0001	0.0023	0.85	0.37
22	lactate /threonine	1.358	0.0029	0.0222	4.01	8.61
25	alanine	1.478	0.0061	0.0415	10.17	12.57
40	glutamate	2.072	0.0008	0.0091	1.51	1.05
42	glutamate	2.147	0.0001	0.0032	1.99	1.29
45	unknown	2.291	0.0032	0.0222	0.28	0.38
48	succinate	2.407	0.0001	0.0022	1.52	3.09
49	glutamine	2.448	0.0031	0.0222	0.77	0.53
50	glutamine	2.480	0.0031	0.0222	0.45	0.31
53	beta-alanine	2.587	0.0005	0.0068	0.23	0.44
59	aspartate	2.834	0.0014	0.0154	0.12	0.08
61	unknown	2.893	0.0007	0.0085	0.07	0.02
62	asparagine	2.937	0.0014	0.0154	0.47	0.28
65	unknown	3.072	0.0018	0.0174	0.10	0.17
74	beta-D-glucose	3.423	0.0000	0.0009	1.37	0.66
75	beta-D-glucose	3.478	0.0003	0.0058	1.74	1.16
82	alpha-D-glucose/B-D-glucose	3.742	0.0000	0.0009	1.83	0.71
88	phenylalanine	3.975	0.0001	0.0028	1.51	1.18
118	trehalose	5.185	0.0022	0.0187	0.54	0.82
120	alpha-D-glucose	5.272	0.0003	0.0058	0.53	0.21
138	5'-UMP	5.999	0.0019	0.0174	0.30	0.20
170	tryptophan/unknown	7.248	0.0000	0.0009	0.32	0.07
178	tryptophan	7.577	0.0007	0.0085	0.37	0.13

180	tryptophan	7.657	0.0004	0.0068	0.29	0.07
191	5'-UMP	8.096	0.0015	0.0155	0.38	0.21

Mean represents the peak area

Table S12. Summary of metabolic response of WT and *fmo* mutants strains across ageing. The observed response of each metabolite at day 6 and 9 post-hatching stages relative to day 3 post-hatching stage (see Tables 1-3 for HMDB numbers).

**Metabolites**	**WT**	***fmo-1* KO**	***fmo-2* KO**	***fmo-2* OE**	***fmo-3* KO**	***fmo-4* KO**	***fmo-4* KO**	**WT**
	**Day 6 vs Day 3**						**Day 9 vs Day 3**	
**Leucine**							↓	
**alpha-D-glucose**	↑	↑		↑		↑	↑	↑
**beta-D-glucose**	↑	↑		↑		↑	↑	↑
**Trehalose**				↓	↓	↓	↓	↓
**Alanine**	↓			↓			↓	↓
**beta Alanine**	↓	↓		↓	↓	↓	↓	↓
**Lactate**	↓			↓			↓	↓
**Threonine**				↓	↓		↓	↓
**Glutamate**				↑			↓	
**Glutamine**				↑			↓	
**Succinate**	↓		↓	↓	↓	↓	↓	↓
**Citrate**							↓	
**Cystathionine**	↓					↓	↓	↓
**Agmatine**	↓		↓		↓		↓	↓

**Choline**							↑	
**Asparagine**	↓			↑	↑		↓	↓
**Aspartate**				↑				↑
**Phosphorylcholine**							↑	
**Trimethylglycine**							↑	
**Tryptophan**				↑	↑		↑	
**Phenylalanine**							↓	↓
**Ethanol**							↑	
**Methanol**							↑	
**Histidine**	↓					↓	↓	↓
**Formate**							↑	
**5′-AMP**	↓					↓	↓	↓
**5′-GMP**							↓	
**5′-ATP**	↓						↓	↓
**5′-UMP**	↓			↑		↓	↓	

increase	decrease

Footnotes: 5'-AMP, 5'-adenosine monophosphate; 5'-ATP, 5'-adenosine triphosphate; 5'-UMP, 5'-uridine monophosphate; 5'-GMP, 5'-guanosine monophosphate. The discriminating metabolites were determined using ANOVA.
